# ATF6α regulates morphological changes associated with senescence in human fibroblasts

**DOI:** 10.18632/oncotarget.11505

**Published:** 2016-08-22

**Authors:** Clémentine Druelle, Claire Drullion, Julie Deslé, Nathalie Martin, Laure Saas, Johanna Cormenier, Nicolas Malaquin, Ludovic Huot, Christian Slomianny, Fatima Bouali, Chantal Vercamer, David Hot, Albin Pourtier, Eric Chevet, Corinne Abbadie, Olivier Pluquet

**Affiliations:** ^1^ Université de Lille, Institut Pasteur de Lille, CNRS UMR8161, Mechanisms of Tumourigenesis and Targeted Therapies, Lille, France; ^2^ Université de Lille, Institut Pasteur de Lille, INSERM, CNRS UMR8204, Centre d'Infection et d'Immunité de Lille, Lille, France; ^3^ Université de Lille, INSERM U1003, Villeneuve d'Ascq, France; ^4^ Oncogenesis, Stress, Signaling, INSERM ER-440 Université de Rennes, Rennes, France; ^5^ Centre de Lutte Contre le Cancer Eugene Marquis, Rennes, France; ^6^ Department of Anatomy and Neuroscience, Biosciences Institute, University College Cork, Cork, Ireland; ^7^ Institut du Cancer de Montréal, Montréal, QC, Canada

**Keywords:** senescence, unfolded protein response, ATF6α, endoplasmic reticulum, normal human dermal fibroblast, Gerotarget

## Abstract

Cellular senescence is known as an anti-tumor barrier and is characterized by a number of determinants including cell cycle arrest, senescence associated β-galactosidase activity and secretion of pro-inflammatory mediators. Senescent cells are also subjected to enlargement, cytoskeleton-mediated shape changes and organelle alterations. However, the underlying molecular mechanisms responsible for these last changes remain still uncharacterized. Herein, we have identified the Unfolded Protein Response (UPR) as a player controlling some morphological aspects of the senescent phenotype. We show that senescent fibroblasts exhibit ER expansion and mild UPR activation, but conserve an ER stress adaptive capacity similar to that of exponentially growing cells. By genetically invalidating the three UPR sensors in senescent fibroblasts, we demonstrated that ATF6α signaling dictates senescence-associated cell shape modifications. We also show that ER expansion and increased secretion of the pro-inflammatory mediator IL6 were partly reversed by silencing ATF6α in senescent cells. Moreover, ATF6α drives the increase of senescence associated-β-galactosidase activity. Collectively, these findings unveil a novel and central role for ATF6α in the establishment of morphological features of senescence in normal human primary fibroblasts.

## INTRODUCTION

After a limited number of divisions, normal cells enter a cell cycle arrest state called senescence, while remaining viable and metabolically active [1]. Senescent cells accumulate in aging organisms and contribute to life span and to some age-related dysfunctions, including lordokyphosis, sarcopenia, fat loss, cardiac arrhythmias, arterial wall stiffening, and dermal thinning [2, 3]. Various mechanisms can induce senescence, including telomere shortening, DNA damage, oxidative stress or oncogene activation [4, 5]. In addition to be cell cycle-arrested, senescent cells exhibit epigenetic and transcriptomic changes, increase in autophagy, Senescence-Associated β-galactosidase (SA-β-Gal) activity, and pro-inflammatory secretome [5, 6]. Several complex signaling pathways related to stress are involved in the induction and/or maintenance of senescence. The most investigated of these pathways is the DNA Damage Response that leads to p53/p21^WAF1^ activation and the senescence-associated irreversible cell cycle arrest in the G1 phase [4]. The cell-cycle inhibitor p16^INK4^/Rb is also robustly activated, in part in response to the oxidative stress which increases at senescence [7, 8]. We and others pointed out Cyclooxygenase 2 (COX-2)-prostaglandin, mTOR and p38/MAPK pathways to participate in senescence [8, 9, 10, 11]. Senescent cells also exhibit profound morphological changes including increase in cell size, in spreading on the substratum and change in cell shape. These morphological changes have been linked to cytoskeletal proteins such as vimentin and to proteins that link cell adhesion to cytoskeleton (e.g. ILK) [12, 13, 14, 15]. It is also thought that increase in senescent cell size may be in part attributed to a decrease in the rate of protein degradation [15]. However, it is likely that other unknown mechanisms participate in morphological, cellular and molecular changes of the senescent phenotype.

In the present study, using the Connectivity Map [16], a database that allows identifying connections between drugs and gene patterns, we provide evidence that the Unfolded Protein Response (UPR) is linked to senescence. The UPR is a complex signaling pathway emanating from three Endoplasmic Reticulum (ER) stress sensors PERK, α) and aims at restoring ER homeostasis [17]he activated PERK kinase phosphorylates its substrate eIF2α to reduce the overall synthesis of proteins, but paradoxically also induces the transcription of genes encoding specific chaperone containing micrORF or IRES. Once activated, IRE1α induces the unconventional splicing of XBP1 mRNA, allowing the translation of an active transcription factor, whose main targets are the genes involved in quality control of proteins in the ER. Other mRNAs are cleaved by IRE1α, however, the physiological consequences associated with this mechanism are not yet fully known. The protein ATF6α, upon activation, exits the ER and migrates to integrate the Golgi apparatus membrane, where it is cleaved to give its active form and acts as a transcription factor targeting genes encoding chaperones. is not resolved, or [18]. ER stress is involved in many diseases, including age-related pathologies such as neurodegenerative disorders and rheumatoid arthritis [19] but little is known about its role in senescence (reviewed in [20]). Our work demonstrates the importance of the UPR in senescence of normal human dermal fibroblasts (NHDFs) and in particular the crucial role of ATF6α in the control of senescence morphological features. Our results suggest that the ATF6α branch of the UPR may represent a potential therapeutic target against senescence-associated cellular dysfunctions in the context of aging.

## RESULTS

### Connectivity map analysis suggests ER stress signaling as a senescence component

To better define the molecular bases of cellular senescence, a gene expression analysis was carried out on senescent vs. exponentially growing NHDFs and a list of genes differentially expressed by at least 1.5-fold (*p* < 0.05) was established (Supplementary Table S1). We first analyzed this senescence gene signature for over-represented Gene Ontology terms and functional assignments (KEGG, PANTHER and Ingenuity Pathway Analysis) (Supplementary Table S2 Excel File). The results revealed only already well-known and classical processes/pathways involved in senescence such as cell cycle regulation and inflammation. To highlight new pathways that could be involved in senescence, the above-mentioned senescence-associated profile was analyzed using the Connectivity Map (CMap) (www.broad.mit.edu/cmap/) [16]. CMap is an alternative approach to associate biological processes with gene expression profiles. It compares the gene patterns of interest, here the senescent signature, with a database of gene patterns produced in cultured human cells treated with so-called perturbagens, i.e. bioactive small molecules. The CMap analysis identified multiple drugs having expression signatures that share similarities with that of senescence. Table 1 shows the top 25 of the compounds with a significant positive enrichment with a *p*-value < 0.05. Interestingly, 7 out of those 25 compounds are known to induce ER stress and/or ER membrane reorganization.

**Table 1:** Pharmaceutical perturbagens with significant enrichment in the senescent gene signature found by Connectivity Map analysis.

Rank	Compound name	Enrichment factor	*p*	Drug category	Known action on ER	ref
1	**Clotrimazole**	0,914	0,00004	antifungal and antimalarial agent,	UPR inducer, induces ER membrane reorganisation	[22] [23]
2	**Puromycin**	0,879	0,0003	Protein synthesis inhibitor	UPR inducer	[21]
3	Meglumine	0,856	0,00056	antiprotozoaires		
4	Colecalciferol	0,842	0,00101	=vitamin D3		
5	Trichlormethiazide	0,84	0,00103	diuretic		
6	Felodipine	0,667	0,00122	calcium antagonist		
7	Trifluridine	0,798	0,00318	antiviral		
8	Lycorine	0,703	0,00547	decreases HDAC enzymatic activities		
9	**Diazoxide**	0,701	0,00573	a potassium channel opener	UPR modulator	[39]
10	Digitoxigenin	0,742	0,00851	cardioactive alkaloids		
11	Pyrimethamine	0,677	0,00893	pro-apoptotic antifolate drug		
12	Anisomycin	0,735	0,00979	Protein synthesis inhibitor.		
13	Laudanosine	0,726	0,0112	neuromuscular-blocking drugs		
14	Pseudopelletierine	0,701	0,01639	alkaloid		
15	Meclozine	0,631	0,01967	antihistaminic		
16	**Pyrvinium**	0,579	0,01984	Anthelminthic	UPR modulator, induces ER membrane reorganisation	[40] [23]
17	Cicloheximide	0,684	0,02148	Protein synthesis inhibitor		
18	**Chloroquine**	0,679	0,02298	anti-malaria drug	UPR inducer	[41]
19	Naftifine	0,676	0,02409	allylamine antifungal drug		
20	**Emetine**	0,672	0,0252	Protein synthesis inhibitor	UPR inducer	[42]
21	folic acid	0,657	0,03209	=vitamin B9		
22	Vincamine	0,547	0,03311	vasodilatator		
23	Bucladesine	0,539	0,03687	cell permeable cAMP analog		
24	**Bupropion**	0,647	0,03724	antidepressant	UPR inducer	[43]
25	**Ivermectin**	0,574	0,04468	parasiticide,	induces ER membrane reorganisation	[23]

The compounds are ranked based on the value of the enrichment factor. Compounds having relationship with ER perturbation are highlighted in bold.

### ER stressors induce premature senescence in normal fibroblasts

**Figure 1:**
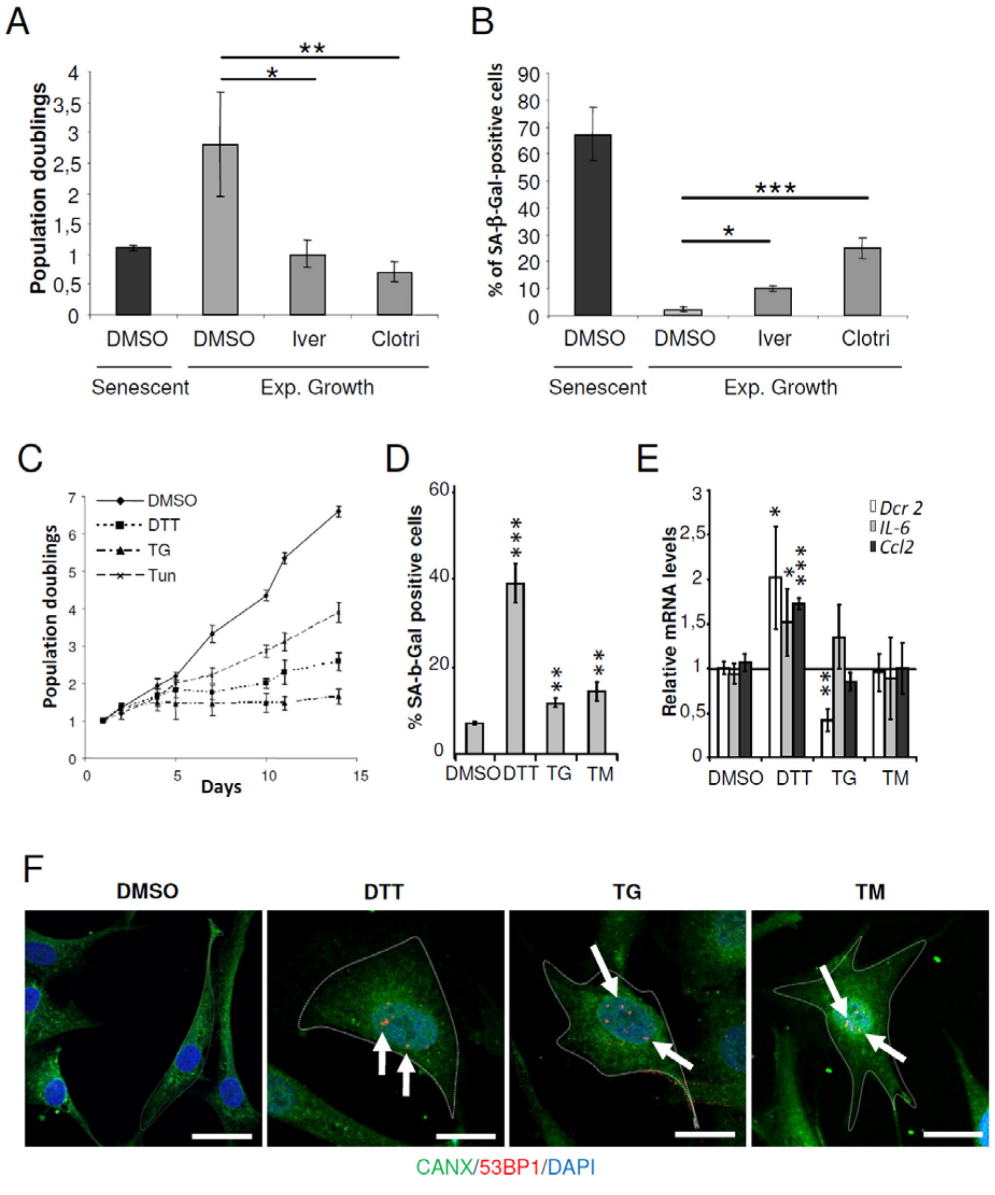
ER stressors accelerated the senescent phenotype in NHDFs **A.** Exponentially growing NHDFs were treated with clotrimazole (10μM) or ivermectin (2μM) for 4 days. The proliferation rate was determined by cell counting. Each condition was tested in triplicate. The bar chart represents the mean ± SD of 3 independent experiments. **B.** Exponentially growing NHDFs were treated as in (A) and the percentage of SA-β-Gal-positive cells was determined. SA-β-Gal positive cells were counted in 3 different microscopic fields. The bar chart represents the mean ± SD of the 3 counts. **C.** Exponentially growing NHDFs were treated with DTT (200 μM), TG (0.1 μM), or TM (0.005 μM), and counted up to 10 days after the beginning of the treatment and cumulative doubling numbers were calculated. Results are representative of three independent experiments performed in triplicate. **D.** Exponentially growing NHDFs were treated with DTT (200 μM) for 4 days, TG (0.1 μM) for 3 days, or TM (0.01 μM) for 3 days and the percentage of SA-β-Gal-positive cells was determined. SA-β-Gal positive cells were counted in 3 different microscopic fields. The bar chart represents the mean ± SD of the 3 counts. **E.** Exponentially growing NHDFs were treated as in (D) and the senescent markers DCR2, IL6, CCL2 were measured at the mRNA levels by qRT-PCR and were normalized to EAR levels. The measures were performed in triplicate. The bar chart represents the mean ± SD of three independent experiments. **F.** Exponentially growing NHDFs grown on coverslips were treated with DTT (200 μM) for 4 days, TG (0.1 μM) for 3 days, or TM (0.005 μM) for 4 days, fixed, and processed for immunofluorescence detection of endogenous protein CANX (*green*) and 53BP1 (*red*). Cell nuclei were detected by DAPI staining (*blue*). Each condition was tested in triplicate. Representative confocal photomicrographs of CANX inmmunostaining and 53BP1 foci (white arrows) are shown. Bars represent 50 μm.

To validate CMap predictions, we tested whether treatment of exponentially growing NHDFs with 2 out of the 7 compounds related to ER stress or ER membrane, clotrimazole and ivermectin [22, 23], could promote premature senescence at the concentrations indicated in CMap. Clotrimazole and ivermectin treated cells showed significantly decreased cell proliferation and significant increase in SA-β-Gal positive cells compared to untreated cells (Figures 1A and 1B). In the same line of idea, we next investigated whether well-known ER stress inducers could promote premature senescence. To this end, exponentially growing NHDFs were treated with thapsigargin (TG), a SERCA inhibitor, tunicamycin (TM), an inhibitor of N-linked glycosylation, or dithiothreitol (DTT), a reducing agent. We first performed dose-responses to determine the non-lethal concentrations of these drugs on growing NHDFs using sulforhodamine B staining (Supplementary Figure S1A). We then verified that the treatments indeed induced ER stress at the selected sub-toxic dose and time. As expected, all treatments significantly induced Bip/GRP78 expression (Supplementary Figure S1B). Next, we examined whether the drug-treated cells acquired senescent characteristics. Exponentially growing NHDFs treated with DTT, TG or TM underwent a reduction of the proliferation rate or even a sustained arrest compared to untreated cells (Figure 1C). All the treatments also significantly increased the percentage of cells displaying SA-β-Gal activity, but DTT was the most effective in doing so (Figure 1D). Moreover, only DTT was able to induce the up-regulation of the senescent markers IL6, CCL2 and DcR2 (TNFRSF10D) (Figure 1E). Immunofluorescence-based detection of Calnexin (CANX), an ER marker, revealed that upon ER stress, cells underwent enlargement. Interestingly, a co-detection of 53BP1 revealed an increased presence of 53BP1 foci inside the nuclei of enlarged cells (Figure 1F). Therefore, under non-lethal conditions, the ER stressors tested in our experiments induced several features of premature senescence including proliferation arrest, SA-β-Gal activity, enlargement and DNA damage.

### Replicative senescence in NHDFs is associated with ER expansion and overexpression of ER-resident proteins

**Figure 2:**
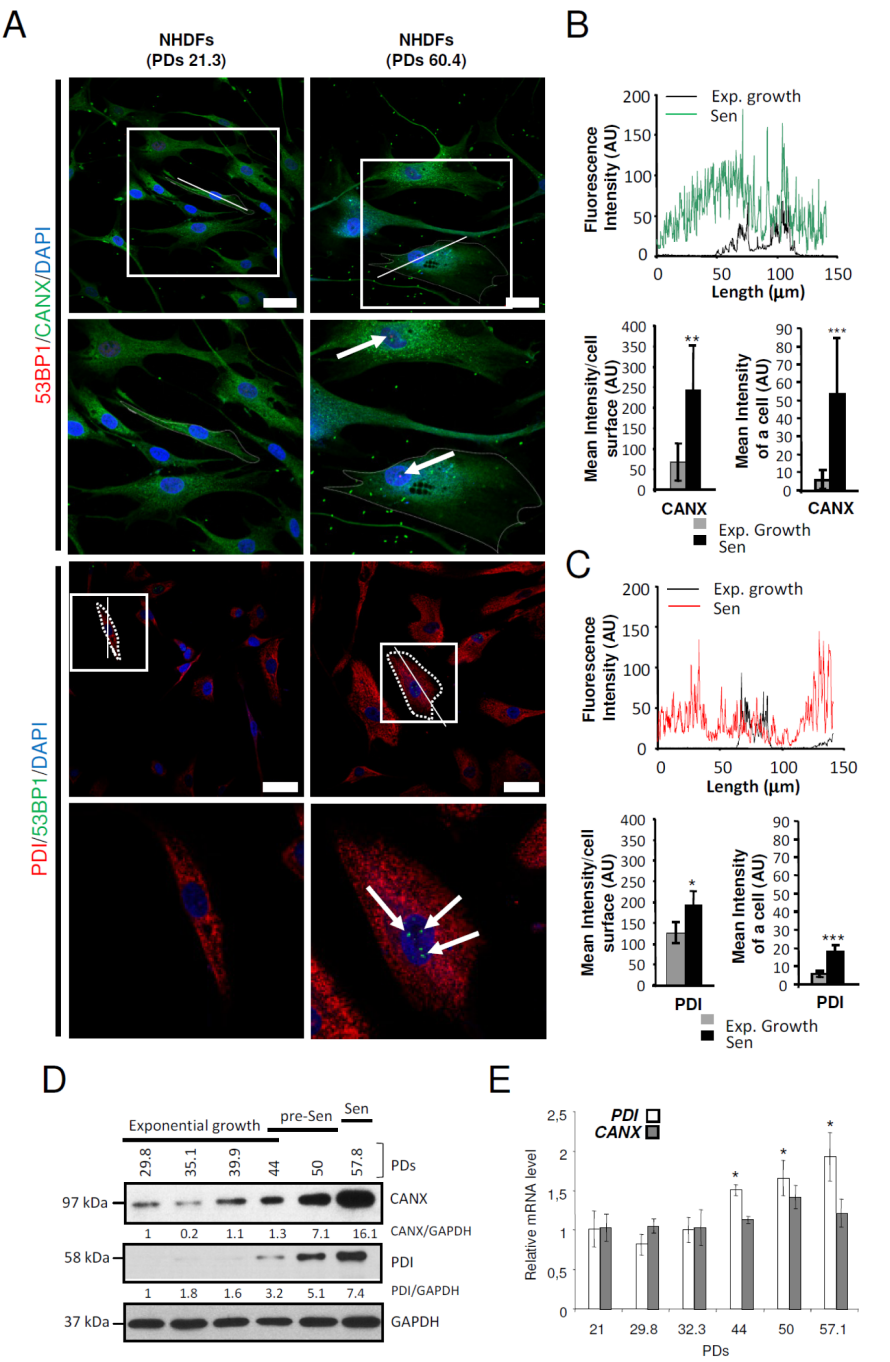
ER-resident proteins are overexpressed at senescence in NHDFs **A.** Exponentially growing (PDs 21.3) and senescent NHDFs (PDs 60.4) were seeded onto coverslips, fixed, and processed for immunofluorescence co-detection of endogenous Calnexin (CANX) (*green*) and 53BP1 (*red*) or Protein Disulfide Isomerase (PDI) (*red*) and 53BP1 (*green*) Cell nuclei were detected by DAPI staining (*blue*). Each condition was tested in triplicate. Representative confocal photomicrographs of CANX immunostaining and 53BP1 foci (white arrows) or PDI immunostaining and 53BP1 foci (white arrows) are shown. Bar = 50 μm. The square delimits the below image at higher magnification. **B.** and **C.** Graphs showing the distribution of fluorescence intensity (arbitrary units) of representative exponentially growing and senescent NHDFs along the largest cellular axis (white line in A). Quantification of the fluorescence signal coupled to anti-CANX antibody (B) or coupled to anti-PDI antibody (C), as mean intensity of a cell, and mean intensity/cell surface, using Image J software. Values are means of >40 cells ± SD. This experiment is representative of 3 independent ones. **D.** Total protein extracts from NHDFs at the indicated population doublings were analyzed by immunoblotting with anti-CANX or anti-PDI antibodies. Expression of GAPDH was used as loading control. Bands were quantified using Image J software. The results are given as a ratio to GAPDH. **E.** PDI and CANX and mRNA levels were measured by qRT-PCR in NHDFs at the indicated population doublings and were normalized to EAR levels. Measures were performed in triplicate. The bar chart represents the means ± SD of the three measures.

In NHDFs, senescence is characterized by a proliferation arrest (Supplementary Figure S2A), huge increase in cell size (Supplementary Figure S2B and C), loss of both the fusiform shape and the parallel organization of the cells associated with cell spreading on the substratum (Supplementary Figure S2B), accumulation of SA-β-Gal positive cells (Supplementary Figure S2D), activation of p53 and up-regulation of p16 (Supplementary Figure S2E). Since the ER generally occupies most of the cytoplasm volume in a cell, we wanted to determine the ER size and structure in the so enlarged senescent cells. To this end, we stained the ER of senescent (PD: 59.7) versus exponentially growing (PD: 32.3) NHDFs using the ER-selective ER-ID fluorescent probe. We first quantified the total fluorescence intensity of ER-ID per cell. The results show that it dramatically increases at senescence, suggesting an ER expansion. However, when this total fluorescent intensity was reported to the surface of the cell, no significant difference was recorded between proliferating and senescent cells. Similarly, the fluorescent intensities measured along the largest cellular axis were similar in proliferating and senescent cells (Supplementary Figure S3A). Therefore, the ER expansion at senescence parallels the cell enlargement. We then determined the impact of senescence on the expression and distribution of the ER-resident proteins Calnexin (CANX) and Protein Disulfide Isomerase (P4HB/PDI) using immunostaining. For both CANX and PDI, we recorded an increase in the total intensity of immunostaining and in the intensity reported to cell size, indicating that ER-resident proteins are overexpressed at senescence (Figures 2A-2C). This overexpression was confirmed by western-blotting experiments in which we observed a progressive and strong increase of CANX and PDI protein levels as soon as the presenescent stage (Figures 2D). In addition, qRT-PCR experiments show that although CANX mRNA level does not vary significantly, PDI mRNA level increased progressively from the pre-senescent to the senescent stage (Figures 2E). Since CANX and PDI have chaperone and enzymatic activities, their increase in expression at senescence could be indicative of ER alterations at senescence. Nevertheless, an ultrastructural analysis by transmission electron microscopy did not reveal any obvious structural change of the rough ER such as dilatation in senescent NHDFs (Supplementary Figure S3B). Taken together, these results provide quantitative evidence supporting an expansion of ER size and an increase in content of some ER-resident proteins at senescence.

### Replicative senescence in NHDFs is associated with activation of the unfolded protein response

**Figure 3:**
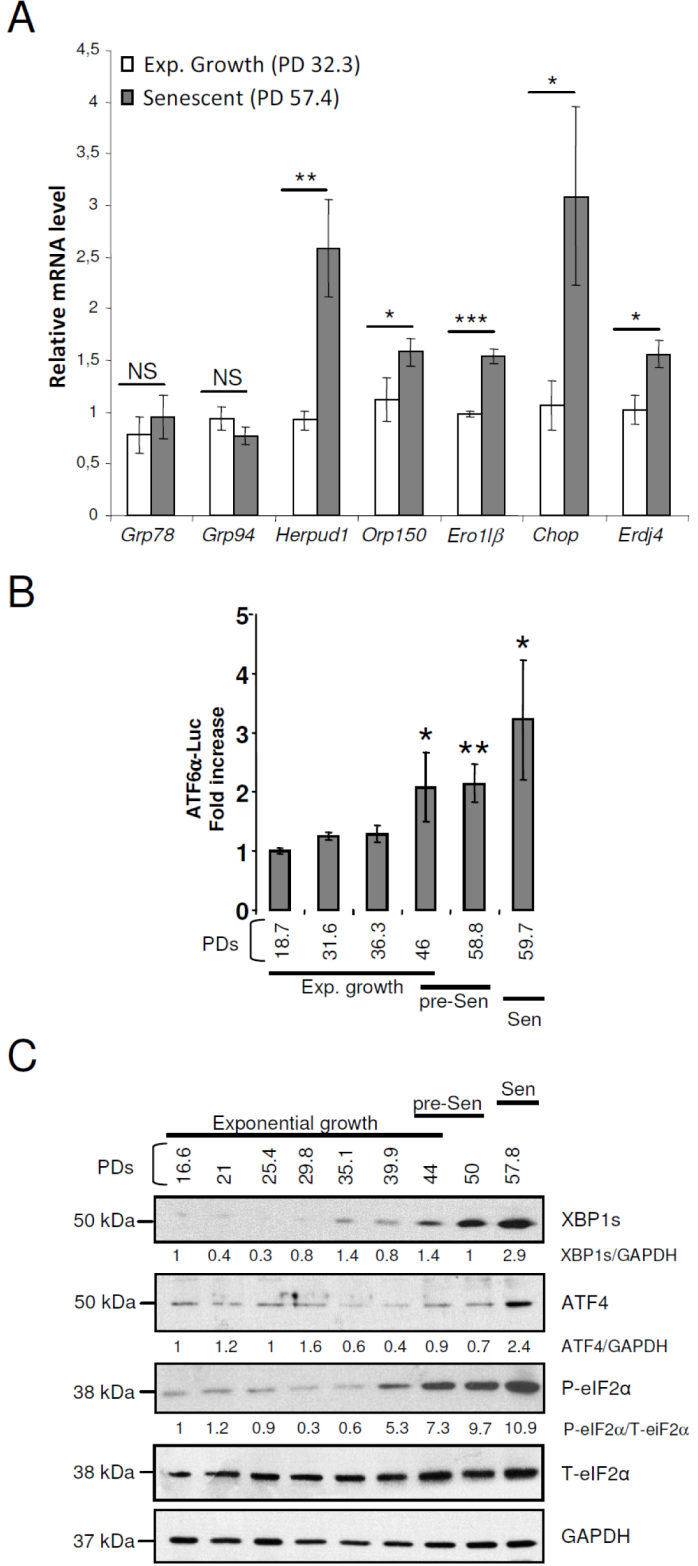
The arms of the UPR are activated with different kinetics in senescent NHDFs **A.** Grp78, Grp94, Herpud1, Orp150, Ero1lβ, Chop and Erdj4 mRNA levels were measured by qRT-PCR and were normalized to EAR levels. Measures were performed in triplicate. The bar chart represents the means ± SD of the three measures. This experiment is representative of 3 independent ones. **B.** Measure of ATF6α transcriptional activity in NHDFs at the indicated population doublings. Each measure was done in triplicate. Each bar represents mean ± SD of the three measures. The results are representative of three independent experiments. **C.** Total protein extracts from NHDFs at the indicated population doublings were analyzed by immunoblotting with anti-XBP1s, anti-ATF4, and anti-Phospho-eIF2α antibodies. Expression of total eIF2α and GAPDH was used as loading controls. The bands were quantified by Image J software. The results are given as the ratio to total eIF2α for Phospho-eIF2α and to GAPDH for XBP1s and ATF4. Results are representative of three experiments.

Alteration of ER size and content in senescent cells may reflect an ER stress that could activate the unfolded protein response (UPR). To monitor the UPR activation status, we measured the induction of a panel of UPR target genes downstream of ATF6α (Grp78, Grp94, Herpud1, Orp150), downstream of IRE1α (Ero1lβ, Erdj4), or downstream of PERK (Chop) using qRT-PCR. mRNA levels of all tested genes except Grp94 and Grp78 were upregulated at senescence (Figure 3A). ATF6α DNA binding activity was monitored with a luciferase reporter gene (containing tandem repeats of the ATF6α transcriptional response element). ATF6α transactivation activity significantly increased as soon as the pre-senescent stage (Figure 3B). Moreover, using immunoblotting, we showed that P-eIF2α levels progressively increased from the very beginning of the pre-senescent phase to reach a 10-fold increase at the senescent plateau. The levels of XBP1s also progressively increased from the pre-senescent to the senescent stage. The levels of ATF4 also increased, but latter, once the senescent plateau was established (Figure 3C). Collectively these data suggest that the UPR is progressively activated during acquisition of the senescent phenotype and remains activated once established. We then wondered whether senescent NHDFs were at their maximum of UPR activation, and whether they were still able to further enhance UPR upon an additional ER stress. To answer to this question, we performed dose response and kinetic analyses with two ER stress inducers (TG and DTT) and examined the expression of various UPR genes. Exponentially growing and senescent NHDFs were treated for 16 hrs with doses of DTT ranging from 50 to 500 μM or with doses of TG ranging from 0.01 to 0.1 μM. An UPR response was elicited in both proliferating and senescent NHDFs only at 500μM DTT or 0.1 μM TG (Supplementary Figures S4A and B). The level of the response to the acute stress was similar in proliferating and senescent cells and was much higher than the level of activation at senescence (Supplementary Figures S4A and B). Next, we performed time-course experiments with 500 μM DTT or 0.1 μM TG. In both cases, the peak of genes induction was delayed by about 2 hrs in senescent NHDFs compared to proliferating ones (Supplementary Figures S5A and B). These data suggest that senescence in primary fibroblasts is associated with a mild activation of the UPR and that senescent cells conserve their ability to adapt to further ER stress.

### The UPR controls the establishment of some but not all senescence markers

**Figure 4:**
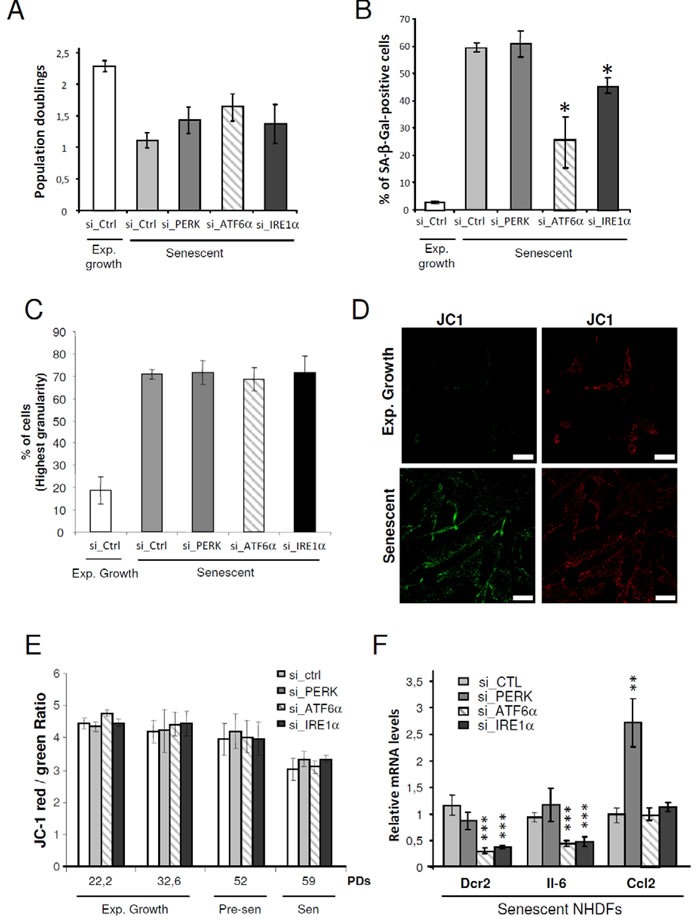
Control of some markers of senescence by the UPR **A.** Exponentially growing or senescent NHDFs were subjected to PERK, ATF6α or IRE1α silencing by siRNA as well as a non-target control. Cells were counted 4 days after the siRNA transfection and the population doublings were calculated. The bar chart represents the mean ± SD of the count of three independent culture dishes. **B.** The percentage of SA-β-Gal-positive cells 4 days after the siRNA transfection is indicated. SA-β-Gal positive cells were counted in 3 independent microscopic fields. The bar chart represents the mean ± SD of each 3 counts. This experiment is representative of 2 independent ones **C.** NHDFs were treated as in (A), counted 4 days after the siRNA transfection, and subjected to flow cytometry analysis to assess their granularity according to their side scatter factor (SSC-A). Bar chart represents the percentage of cells in the subpopulation with the highest granularity. The histograms represent the mean ± SD of the 3 counts. This experiment is representative of 3 independent ones. **D.** Representative confocal microscopic images of JC1 staining performed in exponentially growing and senescent NHDFs. Bar represents 50 μm. **E.** NHDFs were treated as in (A) at different population doubling as indicated, stained with JC-1. A Bar chart representing the JC-1 red/green ratio is given. This experiment represents the mean ± SD of 3 independent measures. **F.** Senescent NHDFs were treated as in A and the senescent markers DCR2, IL6, CCL2 were measured at the mRNA levels by qRT-PCR and were normalized to Rplp0 levels. Results were performed in triplicate. The bar chart represents the mean ± SD of the 3 measures.

To determine whether there is a causal relationship between UPR activation and the onset of senescence, we silenced the expression of each ER stress sensor (PERK, ATF6α, or IRE1α) using RNA interference in NHDFs and examined the impact on the establishment of senescence markers. The efficacy of each siRNAs was verified at both mRNA (Supplementary Figure S6A) and protein (Supplementary Figure S6B) levels four days post-transfection. The protein or mRNA levels of known target genes downstream of each sensor were also measured. The results indicate that each siRNA specifically and efficiently invalidated the targeted UPR sensor and its specific downstream network without affecting the two other UPR branches (Supplementary Figures S6C and S6D). We next evaluated whether invalidating an UPR branch from the beginning of the senescence plateau could alter the establishment of some senescence characteristics, after 4-days post-transfection. Silencing of ATF6α, IRE1α or PERK had no significant effect on cell growth (Figure 4A). However, ATF6α silencing significantly reduced the number of SA-β-Gal positive-cells from about 60% in control cells to 25% in ATF6α silenced NHDFs. A small decrease of SA-β-Gal positive-cells was also observed upon IRE1α knock-down, but not upon PERK knock-down (Figure 4B). As the granules content increased during senescence, we assessed granularity distribution using flow cytometry. The fraction of cells with the highest granularity was increased during senescence. However, silencing of PERK, ATF6α and IRE1α had no impact on this parameter (Figure 4C). Since alteration of the mitochondrial function at senescence was also reported, we next determined whether silencing of the individual UPR sensors could impact on the mitochondrial membrane potential. We analyzed the staining of the membrane permeant JC-1 dye by flow cytometry and observed a significant decrease of the mitochondrial membrane potential in senescent NHDFs compared to proliferating ones (Figure 4D). This decrease was not reverted upon UPR sensor knockdown whatever the population doubling (Figure 4E). We also examined the expression of markers of the secretory pathway by monitoring the mRNA level of genes encoding key secreted components of the Senescence-Associated Secretory Phenotype (SASP), including IL-6 and CCL2 as well as the membrane protein death receptor DcR2 [9]. Silencing of ATF6α and IRE1α led to a drastic decrease in the expression of DcR2 and IL-6 but did not affect the expression of CCL2. In contrast, PERK silencing led to a significant increase in CCL2 level, and did not impact on the expression of the other markers (Figure 4F). Since IRE1α knockdown was less efficient than the knockdown of PERK and ATF6α (Supplementary Figure S6), we also pharmacologically inhibited IRE1α using 4 μ8c or toyocamycin and silenced one of its direct target, XBP1s (Supplementary Figure S7A). These strategies confirmed the results obtained with si_IRE1α treatment, indicative of a weak decrease of SA-β-Gal positive cells (Supplementary Figures S7B), but of no effect on cell proliferation, granularity or mitochondrial depolarization (Supplementary Figures S7C, D and E). These results suggest that ATF6α and, to a lesser extent, IRE1α control part of the increase in SA-β-Gal activity at senescence and are required for the transcriptional induction of gene coding for transmembrane and selected secreted proteins.

### ATF6α activation controls the cell shape associated to senescence

**Figure 5:**
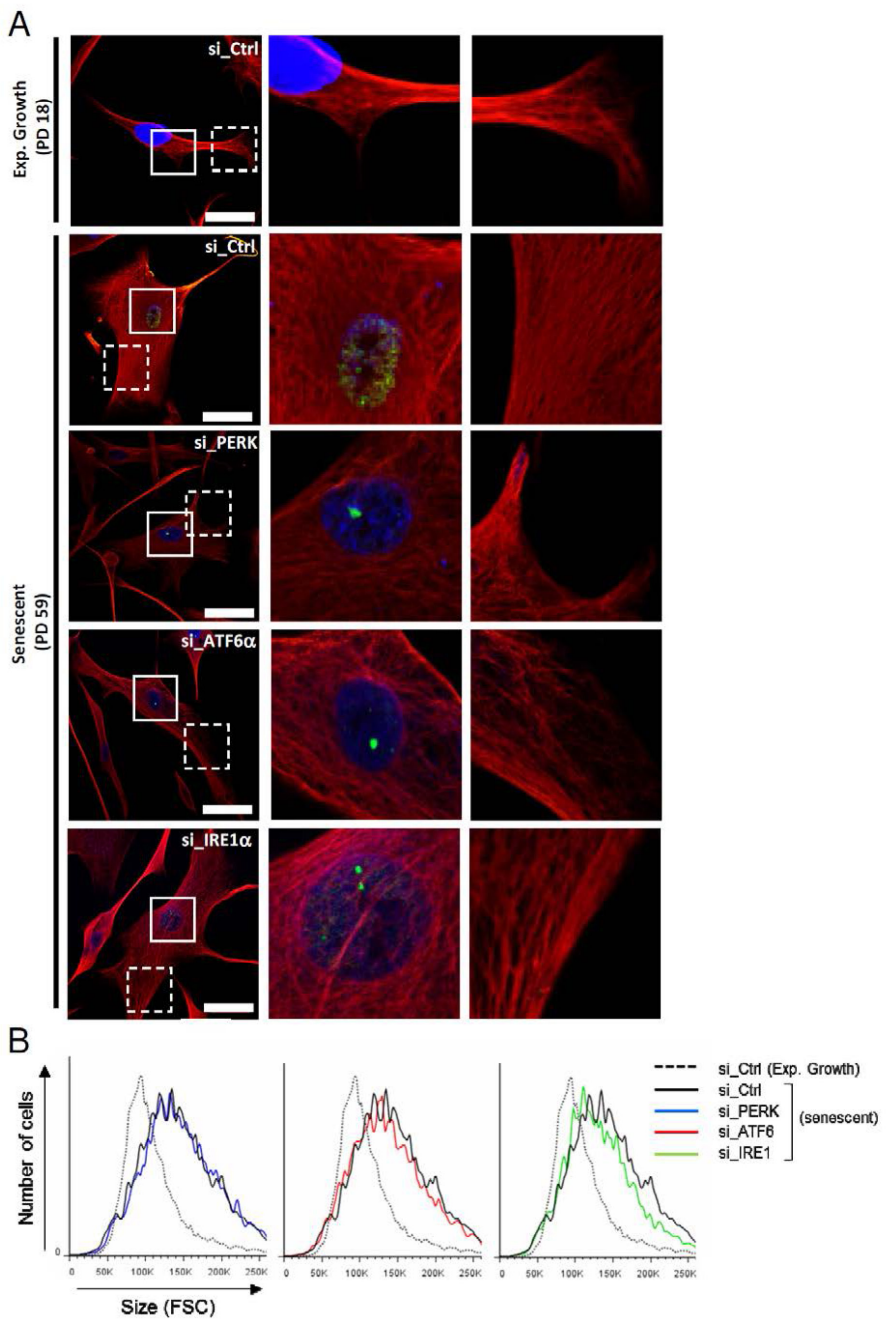
Cytoskeleton mediated-morphological changes of senescent NHDFs are dependent of ATF6α **A.** Senescent NHDFs were subjected to PERK, ATF6α or IRE1α silencing by siRNA as well as a non-target control. Representative confocal photomicrographs of endogenous vimentin (*red*) inmmunostaining and 53BP1 foci (*green)* are shown. Cell nuclei were detected by DAPI staining (*blue*). Bars represent 50 μm. Middle panels, images at higher magnification to visualize 53BP1 foci. Right panels, images at higher magnification to visualize vimentin organization. **B.** Exponentially growing and senescent NHDFs subjected to PERK, ATF6α or IRE1α silencing by siRNA as well as a non-target control and exponentially growing NHDFs were analysed by flow cytometry. Cell distribution was plotted against cell size (forward scattering, FSC).

Since the ER expansion associated with senescence is correlated with the senescent cell enlargement, we next investigated the role of the UPR sensors in cell morphology and size changes associated to senescence. We analyzed the vimentin network by immunofluorescence using confocal microscopy after invalidation of PERK, IRE1α and ATF6α. Interestingly, only ATF6α silencing during senescence led the cells to restore a fusiform shape close to that of exponentially growing cells (Figure 5A). Co-staining of vimentin and 53BP1 indicates that the cells invalidated for ATF6α with a restored fusiform shape displayed 53BP1 foci, indicating that they were initially bona fide senescent cells with DNA damage (Figure 5A). Higher magnifications show that senescent cells present long, dense and often paralleled bundles of vimentin filaments compared to exponentially growing NHDFs. We noticed that silencing of the UPR sensors led to more irregular and less dense networks of vimentin filaments (Figure 5A). We next quantified changes of cell size in suspension using flow cytometry (Figure 5B). Senescent NHDFs exhibited an increase in cell size measured as the FSC value compared to growing cells. Silencing of individual UPR sensors in senescent cells did not restore the normal size of the cells (Figures 5B and Supplementary S8A). This gain in cell size at senescence was correlated with an increase in protein content, which was not significantly changed upon silencing of the UPR sensors (Supplementary Figure S8B). Further experiments with pharmacologic inhibition of IRE1α did not confirm the effect on cell size (Supplementary Figure 9A and B) but showed that cell spreading was reduced (Supplementary Figure S9A). Total cell protein content was not decreased upon inhibition of IRE1α activity but in contrast slightly increased (Supplementary Figure S9C). The invalidation of XBP1 had no impact either in cell shape, size or protein content (Supplementary Figure S9). These data suggest that the UPR could control the changes in cell shape occurring at senescence, mostly through ATF6α.

**Figure 6:**
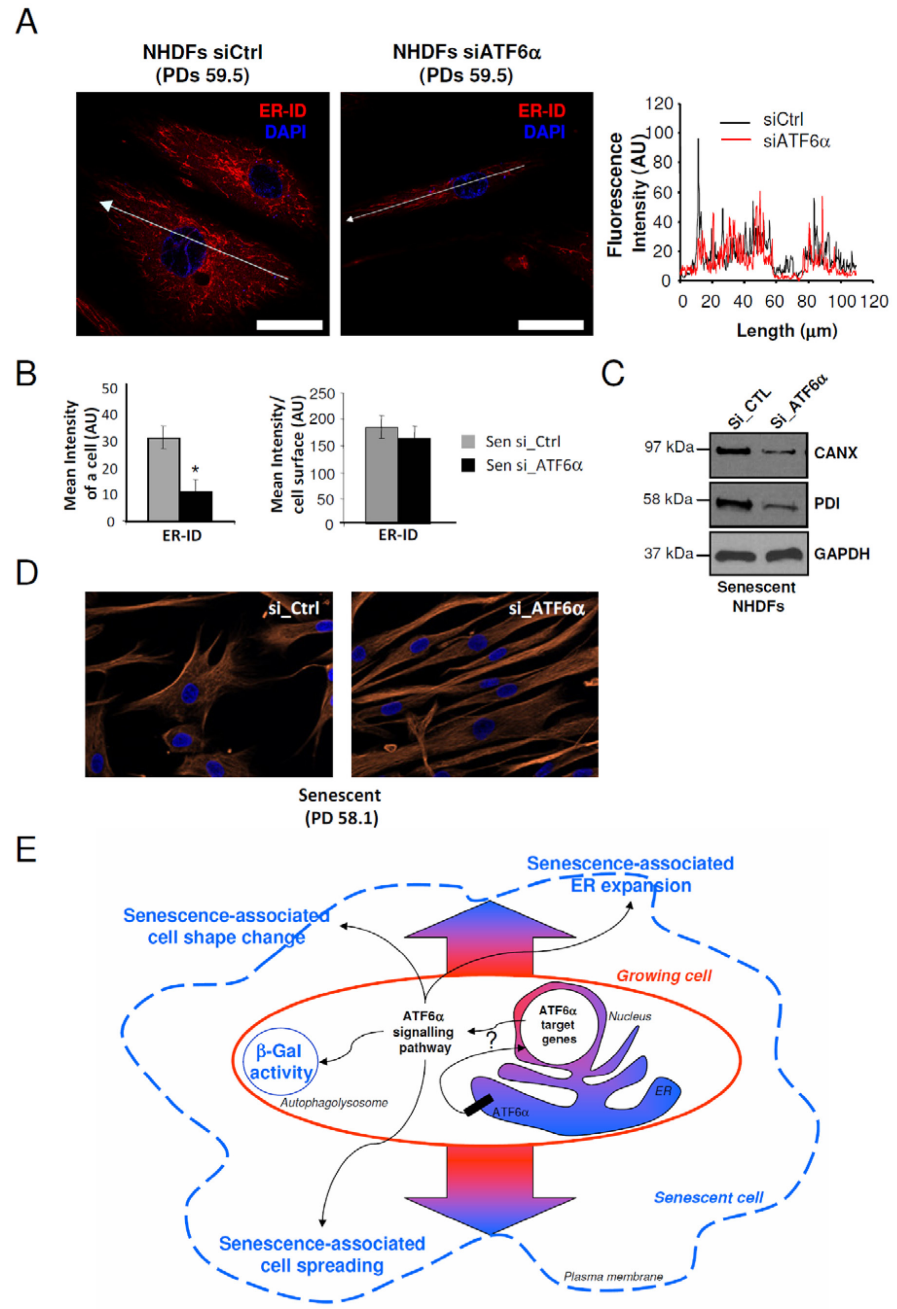
ER expansion and cell shape changes are partly reversed by ATF6α silencing in senescent NHDFs **A.** Senescent NHDFs were subjected to ATF6α silencing by siRNA as well as a non-target control and processed for fluorescence detection of ER using ER-ID (*red*). Cell nuclei were detected by DAPI staining (*blue*). Each condition was tested in triplicate; representative images are shown. Right panel: Graphs show the distribution of fluorescence intensity (arbitrary units) of representative growing and senescent NHDFs along the largest cellular axis (white line in left panels). Bar = 50 μm. **B.** Quantification of the fluorescence signal coupled to ER-ID, as mean intensity of a cell, and mean intensity/cell surface, using Image J software. Values are means of >20 cells ± SD. This experiment is representative of 3 independent ones. **C.** Senescent NHDFs were subjected to ATF6α silencing by siRNA as well as a non-target control. Total protein extracts were analyzed by immunoblotting with anti-CANX and anti-PDI antibodies. Expression of GAPDH was used as loading controls. **D.** Senescent NHDFs were subjected to ATF6α silencing by siRNA as well as a non-target control. Immunofluorescence analysis was performed on endogenous vimentin (*red*), and cell nuclei were detected by DAPI staining (*blue*). Each condition was tested in triplicate; representative images are shown. Bar = 50 μm. **E.** Schematic representation of the role of ATF6α signalling in senescence in primary fibroblasts. Normal growing NHDFs (delimited by a red line) can enter replicative senescence (blue line). The ATF6α arm of the UPR is activated during senescence and is thought to be a key player in controlling morphological changes associated to senescence, including cell enlargement, cell shape, ER expansion, and SA-β-Gal activity.

Since changes in cell shape at senescence are associated with ER expansion, we hypothesized that ATF6α could control them by controlling ER expansion. We therefore determined whether silencing of ATF6α in senescent NHDFs could restore the ER size together with restoring a fusiform shape. To this end, we quantified fluorescence intensities of the ER-ID fluorescent probe along the largest cellular axis and found that ER size decreased proportional to the cell surface decrease upon ATF6α knock-down (Figures 6A and 6B). This effect was also associated with a reduction in CANX and PDI expression upon ATF6α silencing (Figure 6C). Moreover, after invalidation of ATF6α, cells displayed not only a restoration a normal less-spread fusiform shape but also a normal parallel organization of the cells (Figure 6D). Taken together, these results indicated that ATF6α may play a major role in cell shape and ER homeostasis associated to the senescent phenotype of NHDFs.

## DISCUSSION

Studies have highlighted an UPR activation in various models of senescence (replicative, oncogene-induced or stress-induced) and in different cell types [20].

During senescence, ER stress is materialized by the expansion of the ER membrane network. ER expansion and biogenesis are known to occur mostly through ATF6α and IRE1α mediated phospholipid biosynthesis and aims to adapt the ER capacity to the protein folding demand [24, 25, 26, 27, 28]. Our experiments of invalidation by siRNAs show that the ER expansion at senescence is also controlled by ATF6α. It could be induced to adapt to an increased demand in protein synthesis, perhaps proteins of the senescent pro-inflammatory secretome, since the invalidation of ATF6α also impacts the production of IL6.

Remarkably, the ATF6α-controlled ER expansion was always coupled in our experiments with changes in cell shape, but not with changes in cell size. Cell shape changes are governed by the interplay of cytoskeleton networks of actin, microtubules and intermediate filaments (review in [29, 30]). Our data suggest that ATF6α-dependent morphological changes at senescence are linked to remodelling of the intermediate filament cytoskeleton of vimentin. An increased vimentin expression leading to thicker, longer and regularly packed bundles was reported in senescent fibroblasts [12, 13, 14]. We confirm here these data and show that these changes in vimentin cytoskeleton are in part under the control of ATF6α. Although the ER itself is anchored to the cytoskeleton (review in [31, 32]), almost nothing is known about activation of UPR signalling and vimentin cytoskeleton remodelling except a study showing that silencing ATF6α lowers the expression of vimentin [33]. Therefore, one can easily propose that activation of the ATF6α signalling at senescence may involve concerted mechanisms between ER and cytoskeleton.

Activation of the UPR upon ER stress primarily aims at restoring ER homeostasis, condition that enables the stressed cells to adapt to the detrimental conditions to which they are exposed and to survive. However, a sustained UPR activation is known to induce apoptosis or other forms of cell death mostly through the PERK/CHOP pathway [34]. Recent data suggest that the relative dynamics between the IRE1α/XBP1s and PERK/ATF4 pathways are important determinants of the cell outcome, with a late activation of IRE1α immediately followed by fast ATF4 accumulation resulting in cell death versus an early activation of IRE1α and late translation of ATF4 resulting in cell survival [35]. In culture, the replicative senescence plateau of NHDFs is very stable and not accompanied by any significant cell death for up to several months. Nevertheless, the proapoptotic PERK/CHOP pathway is activated, but it could be antagonized by the pro-survival effect of the very long delay (about 150 days, 13 population doublings) between activation of the IRE1α/XBP1s axis and ATF4 accumulation. Anyway, the mild and progressive UPR activation that occurs at senescence in fibroblasts does not seem to play a crucial role in the survival/death balance of senescent cells, since the invalidation of neither of the UPR sensor induced the death of senescent cells (data not shown). Activation of PERK has also been implicated in cell cycle regulation through the phosphorylation of eIF2α [36]. However, we did not observe any effect of PERK invalidation on the proliferation of senescent cells

In conclusion, our data provide the first evidence of the involvement of ER stress in the induction of senescence in NHDFs through ATF6α activation. Importantly, ATF6α appears to be a key player that controls the loss of fusiform shape and parallel organization of the cells, the SA-β-Gal activity and ER expansion associated to the senescent phenotype (Figure 6E). Further investigations on selective ATF6α target genes that may influence cell enlargement is required as well as the ATF6α-dependent mechanisms that govern other processes associated to senescence and linked to the functioning of the ER, including autophagy and senescent associated secretory phenotype (SASP). Therefore, targeting specific components of the ATF6α pathway may have potential anti-aging effects.

## EXPERIMENTAL PROCEDURES

### Compounds and cell culture

Normal human dermal fibroblasts (NHDFs) were purchased from Clonetics (Basel, Switzerland) or PromoCell (Heidelberg, Germany). According to the experiment, four different skin donors were used (referred as 1F1853, a 38 years-old Caucasian female, 2F1966, a 37 years-old Caucasian female, F1MC, a 1 year-old Caucasian male, F6MC1, a 6 years-old Caucasian male). Skin donors are anonymous and informed consent of each skin donor was obtained by the supplier. NHDFs were grown at 37°C in an atmosphere of 5% CO_2_ in the *ad hoc* medium supplied by Clonetics (FGM-2 bulletKit system). In all experiments, cells were seeded as recommended by the supplier and always split at 70% confluence. The number of population doublings (PDs) was calculated at each passage by using the following equation: PD=ln(number of collected cells/number of plated cells)/ln2. Depending on the donor, the senescent stage was reached after a slightly different number of population doublings. The exponential growth phase is defined as the phase during which cells divide regularly, do not display the enlarged and spread morphology nor express the SA-β-Gal marker. Cells designated “senescent” were taken at the senescence plateau, a phase during which the growth of the culture is stopped, with 60 to 70% of cells displaying the enlarged and spread phenotype and the SA-β-Gal activity. The SA-β-Gal activity was revealed as described by [37]. Clotrimazole, Ivermectin, Toyocamycin, Thapsigargin (TG), Tunicamycin (TM), Dithiothreitol (DTT) were purchased from Sigma-Aldrich (St. Louis, MO, USA) and diluted in DMSO. Lysotracker and ER-ID were purchased from Molecular probes (Life Technologies, Thermo Fischer Scientific Inc, Rockford, IL, USA) and Enzo Life Sciences (Villeurbanne, France) respectively. 4μ8c was purchased from Merck Millipore (Calbiochem, Darmstadt, Germany). Lysotracker was purchased from Molecular Probes (Life Technologies, Thermo Fischer Scientific Inc, Rockford, IL, USA). Living cells were incubated with the probe directly added to the culture medium at 37°C for 30 min as recommended by the supplier.

### ATF6 DNA binding assay

Cignal Lenti ATF6 Reporter assay (firefly luciferase) and Cignal Lenti TK Renilla Control were purchased from Qiagen (Venlo, Netherlands), the dual-Luciferase Assay system was purchased from Promega (Madison, WI, USA). NHDFs at different population doublings were seeded at a density of 2 x 10^4^ cells per well in 12-well plates and were transduced using the SureENTRY Transduction reagent (Qiagen) according to manufacturer's instructions.

### Western blotting

Equal numbers of cells were lysed in the following solution: Hepes 27.5 mM pH 7.6, urea 1.1 M, NaCl 0.33 M, EGTA 0.1 M, EDTA 2 mM, KCl 60 mM, DTT 1 mM and NP40 1.1%. The total protein content was measured using the bicinchoninic acid method. Proteins were resolved by SDS-PAGE and transferred to nitrocellulose membranes (Hybond-C extra, Healthcare Life Sciences, Piscataway, NJ, USA). Equal loading was verified after a Ponceau Red coloration of the membranes. Primary antibodies used were an anti-human eIF2α (Santa Cruz Biotechnology, Dallas TX, USA), anti-human Phospho-eIF2α (Cell Signalling, Boston, MA, USA), anti-human XBP1s (gift from E Chevet), anti-human CANX (Abcam, Cambridge, UK), anti-human ATF4 (Santa Cruz), anti-human p53 (Santa Cruz), anti-human p16 (BD Biosciences Pharmingen, San Diego, CA, USA), anti-human PDI (Enzo Life Science, Lausanne, Switzerland) or anti human GAPDH antibody (Santa Cruz). Secondary antibodies used were peroxidase-conjugated (Jackson ImmunoResearch Laboratories, West Grove, PA, USA). Peroxidase activity was revealed using an ECL (enhanced chemiluminescence) or ECL advanced kit (GE Healthcare Life Sciences). The band densities were quantified using Image J software. The density of each band was divided by the density of the GAPDH band or related control band, and the obtained value was normalized with respect to that obtained under control conditions.

### RNA isolation, Reverse transcription and quantitative real-time PCRs (qRT-PCR)

Total RNA was prepared using the Trizol reagent (Life Technologies, Carlsbad, CA, USA). One μg of RNA was reverse-transcribed using random hexamers, Superscript III and dNTPs (Life Technologies) in a final volume of 20 μl according to manufacturer's instructions. Quantitative Real-time PCR reactions were performed using the Mx3005P Real-time PCR system (Agilent, Santa Clara, CA, USA). Primers used were designed with the qPrimerDepot software (http://primerdepot.nci.nih.gov/) and are listed in Table S2. PCR products were measured by SYBR Green fluorescence (SYBR Green Master Mix, Life Technologies). Experiments were performed in triplicates for each data point. Results were analyzed with the MxPro software (Agilent). The expression of each gene was normalized to Rplp0 or EAR gene, and fold expression relative to the control is shown.

### Small interference RNA

Invalidation experiments for PERK, ATF6α, IRE1α and XBP1 were performed using pools of four siRNAs from Dharmacon (ON TARGET Plus Smart pool, Dharmacon, Lafayette, LA, USA). A non-targeting siRNA pool (Dharmacon) was used as control. Senescent NHDFs were seeded at a density of 5 x 10^4^ cells per well in 6-well plates and were transfected by using the RNAiMAX Lipofectamine reagent (Life Technologies) according to manufacturer's instructions.

### Immunofluoresence

NHDFs were seeded onto coverslips and fixed with 4% paraformaldehyde in PBS and permeabilized with 0.2% Triton-X-100. Coverslips were incubated with the primary antibody diluted in presence of 5% BSA. They were then washed three times with PBS and incubated with the secondary antibody diluted in PBS with 0.5% casein. After three washes, nuclei were stained with Hoechst 33258 at 1 μg/ml for 3 minutes and the coverslips mounted in Glycergel^®^. Primary antibodies used were rabbit anti-calnexin from Abcam (dilution 1/100), rabbit anti-vimentin from Santa Cruz (dilution 1/100), rabbit anti-PDI (Enzo Life Science), and mouse anti-53BP1 (Santa Cruz). Secondary antibodies were IgG conjugated with Alexa Fluor 488 or Alexa Fluor 555 purchased from Life Technologies. Images were taken with either apotome or a confocal Zeiss LSM780 microscope (Zeiss, Germany). The ZenLite^®^ (Zeiss, Germany) and ImageJ softwares were used for microscope image analysis.

### Transmission electron microscopy

Adherent primary fibroblasts were fixed with 2.5% glutaraldehyde in 0.1 M cacodylate buffer, pH 7.4 for at least 30 min at 4°C. After fixation, the specimens were thoroughly washed in 0.1 M cacodylate buffer and then postfixed with 1% osmium tetroxide in the same buffer for 1h at room temperature, stained with 2% uranyl acetate in distilled water for 15 min, dehydrated in graded acetonitrile, and embedded in Epon. Ultrathin sections (80-90 nm thick) were cut on a Leica UC7 (Leica Microsystems, Nanterre, France), transferred on 150-mesh grids and contrasted with 2% uranyl acetate solution and Reynolds lead citrate solution. The electron micrographs were taken with a Hitachi H600 (Hitachi, Krefeld, Germany) transmission electron microscope at 75 kV accelerating voltage.

### Flow cytometry

Flow cytometric analyses were performed using a Coulter EPICS XL-MCL (Beckman Coulter, Pasadena, CA), a BD LSR Fortessa (Becton Dickinson, Erembodegem, Belgium) or a BD FACSCanto II (Becton Dickinson). Collected data were exported to the FACSDiva 6.0 software (BD Biosciences) to select subpopulations according to their forward and scatter factors.

### Evaluation of the mitochondrial membrane potential

After a wash in PBS, cells were incubated with 1μM of JC-1 dye (Life Technologies, T-3168, Thermo Fischer Scientific Inc) for 30min and washed again. JC-1 staining was analyzed using flow cytometry. For recording the green fluorescence indicative of depolarization, cells were excited at 488nm, and emission was detected using a 530±40nm band pass filter. For recording the red fluorescence indicative of intact *Δψ*(m), cells were excited at 488nm, and emission was detected using a 613±20nm band pass filter.

### Microarray analysis

Microarray analyses were conducted following the Two-Color Microarray-Based Expression Analysis Protocol (Agilent Technologies, Santa Clara, CA, USA). For each sample, 1 μg of total RNA was divided into two equal aliquots to enable technical replication known as ‘dye-swap hybridization’. The reverse transcription and the labelling procedure were performed using the protocol recommended by Agilent Technologies (Low Input Fluorescent Linear Amplification Kit). Hybridizations were performed on Agilent Whole Human Genome 44K microarrays for 17h at 65°C using the Agilent Gene Expression Hybridization kit. Arrays were washed and scanned using Innoscan 700 (Innopsys, Carbonne, France), and the raw data were processed and normalized using the Limma package (Linear Models for Microarray Data) running under the R environment. Only the differentially expressed genes by at least 1.5-fold (*p* < 0.05) in senescent *vs* growing NHDFs were taken in account (Table S1). Three biological replicates (F1MC) at PD59.3 vs PD18.2 were used.

### Connectivity Map

We compared our senescence signature to signatures from the Connectivity Map (CMap) resource (www.broad.mit.edu/cmap/). The 100 most up- and down-regulated genes in the senescence signature were selected. These were then mapped to the U133A probe sets in order to query the online database. The similarity of the query signature to each of the reference expression profiles was assessed and quantified by a normalized score, from −1 for a molecule that reverses the signature to +1 for a molecule that induces gene expression changes similar to the query signature. We selected only compounds for which significant positive enrichment factor was found and for which at least 4 sets of data were entered in the CMap database.

### Sulforhodamine B (SRB) assay

The SRB assay was performed as previously described [38]. Briefly, NHDFs were seeded into 96-well plates at a density of 1,000 cells/well. After cell inoculation, the plates were incubated at 37°C with 5% CO2 for 24 h prior to addition of experimental drugs. A bar chart represented the SRB proliferation assay (% of control cell growth) by using the following formulae: (mean OD sample - mean OD day0)/(mean OD neg control - mean OD day0)x100.

### Statistical analyses

Statistical analyses were done using one-way ANOVA to evaluate the differences among more than three groups, and/or using the Student's *t*-test to evaluate the difference between two groups. The p values are indicated in the diagrams with * for *p* values < 0.05, ** for *p* values < 0.01 or *** for p values < 0.001. When *p* values > 0.05, differences are considered as non-significant.

## SUPPLEMENTARY MATERIALS METHODS, FIGURES AND TABLES





## Abbreviations

UPR: Unfolded Protein Response
ER stress: Endoplasmic Reticulum stress
PERK: Protein kinase RNA-like ER kinase
ATF6α: Activating Transcription Factor 6α
IRE1α: Inositol-Requiring enzyme 1α
XBP1: X-Binding Protein 1
SA-β-gal: Senescence Associated-β-Galactosidase
DTT: Dithiothreitol
TM: Tunicamycin
TG: Thapsigargin
Toyo: Toyocamycin
NHDFs: Normal human dermal fibroblasts
