# Supplementary material for: ATF6α regulates morphological changes associated with senescence in human fibroblasts
Source: Oncotarget. 2016 Aug 22;7(42):67699–715. doi: 10.18632/oncotarget.11505 (PMC5356513; doi:10.18632/oncotarget.11505)
Supplement: Supplementary file 1 [file oncotarget-07-67699-s001.pdf]

ATF6α regulates morphological changes associated with senescence in human fibroblast

Supplementary Material

Supplementary Procedure

Measure of total protein content – Equal numbers of cells were lysed in RIPA buffer containing (in PBS) 0.1 % (w/v) SDS, 0.5 % (w/v) sodium deoxycholate, and 1.0 % (w/v) Nonidet P-40. The total protein content was measured using the bicinchoninic acid method and is expressed as µg protein/10^4 cells.

Western blotting – Equal numbers of cells were lysed in the following solution: Hepes 27.5 mM pH 7.6, urea 1.1 M, NaCl 0.33 M, EGTA 0.1 M, EDTA 2 mM, KCl 60 mM, DTT 1 mM and NP40 1.1%. The total protein content was measured using the bicinchoninic acid method. Proteins were resolved by SDS-PAGE and transferred to nitrocellulose membranes (Hybond-C extra, Healthcare Life Sciences, Piscataway, NJ, USA). Equal loading was verified after a Ponceau Red coloration of the membranes. Primary antibodies used were an anti-human PERK (Santa Cruz Biotechnology, Dallas TX, USA), anti-human ATF6α (Bioacademia, Osaka, Japan), anti-human IRE1α (Santa Cruz) or anti human GAPDH antibody (Santa Cruz). Secondary antibodies used were peroxidase-conjugated (Jackson ImmunoResearch Laboratories, West Grove, PA, USA). Peroxidase activity was revealed using an ECL (enhanced chemiluminescence) or ECL advanced kit (GE Healthcare Life Sciences).

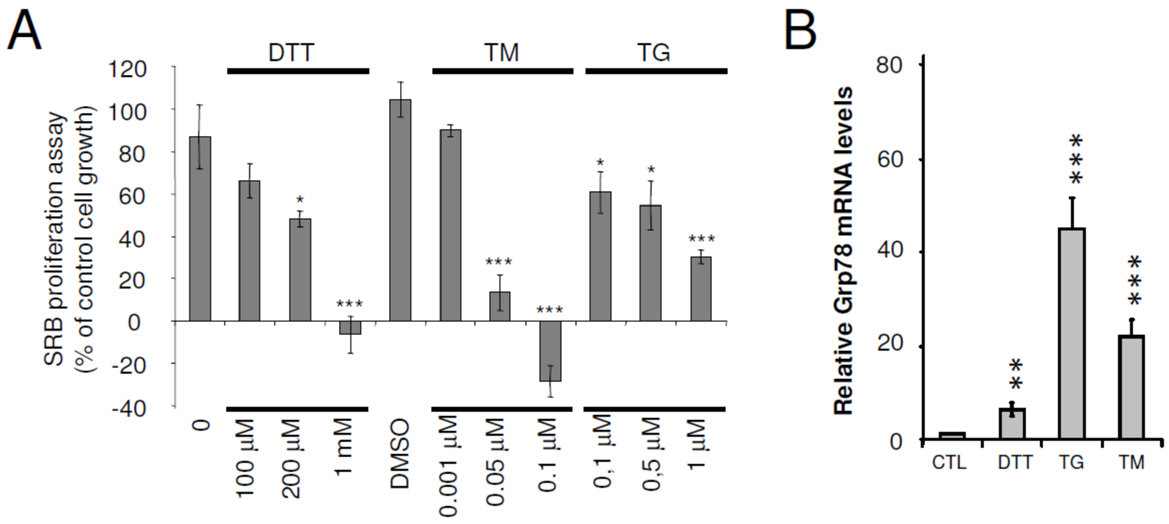

Supplementary Figure S1: Complements to Figure 1. (A) Exponentially growing NHDFs were treated with increasing concentrations of Dithiothreitol (DTT) (100µM, 200µM and 1mM) for 4 days, tunicamycin (TM) (0,001 µM, 0,05 µM and 0.1 µM) for 3 days or thapsigargin (TG) (0,01 µM, 0,5 µM and 1 µM) for 3 days,. The proliferation rate (OD at 510 nm) was determined by using sulforhodamine B (SRB) staining. Each condition was tested in triplicate. The bar chart represents the mean ± SD of 3 independent experiments. Negative values indicate toxicity where the number of viable cells after treatment is lower than the one seeded. (**B**) Exponentially growing NHDFs were treated with DTT (200 µM for 4 days), TG (0.1 µM for 3 days) or TM (0.01 µM for 3 days) and Grp78 mRNA levels were measured by RT-qPCR and were normalized to EAR levels. Measures were performed in triplicate. The bar chart represents the mean ± SD of 3 independent experiments.

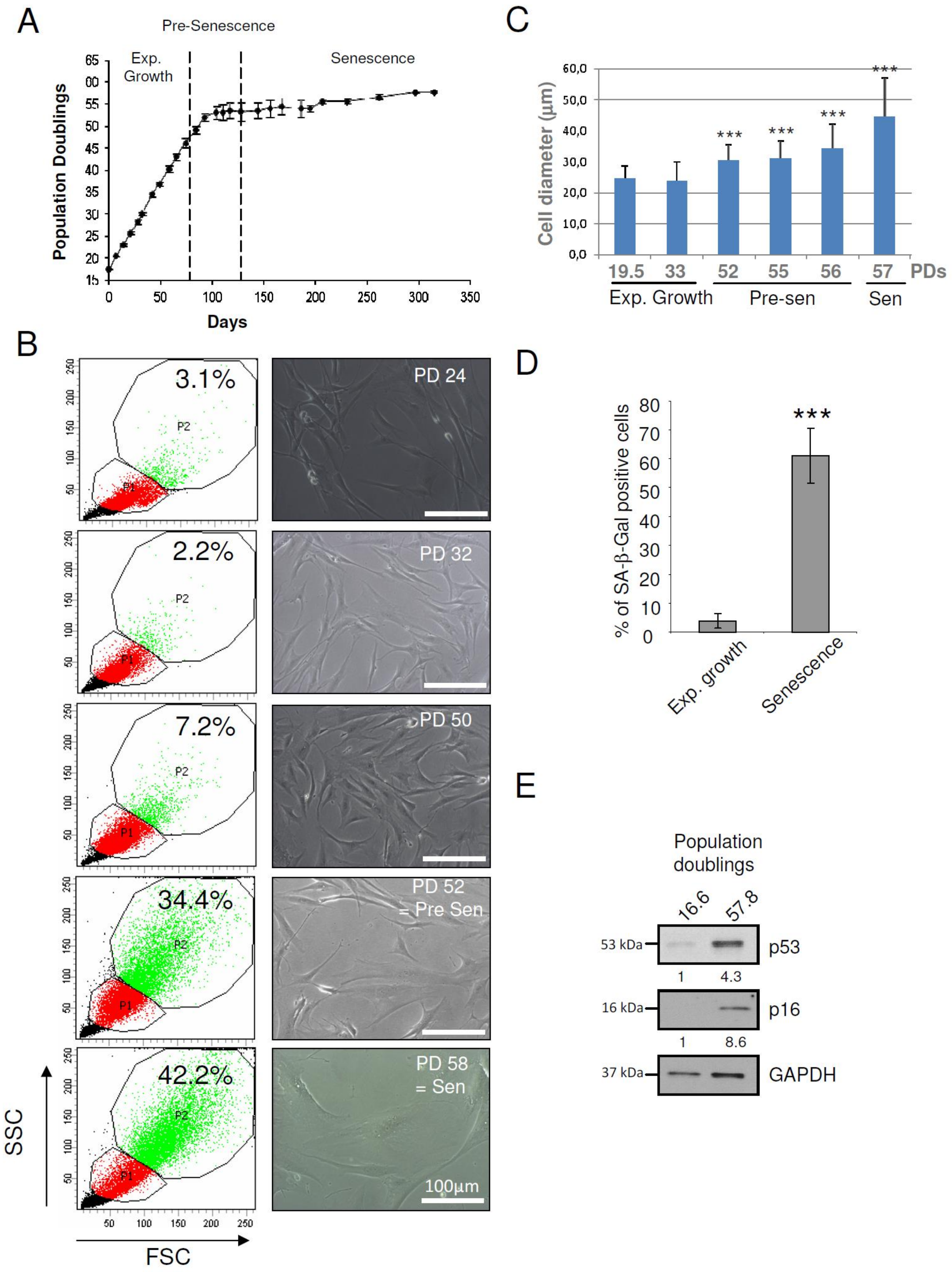

Supplementary Figure S2: Molecular and cellular characterization of replicative senescence of NHDFs. (**A**) Growth curve of NHDFs. (**B**) NHDFs at different population doublings were subjected to flow cytometry analysis to assess their size and granularity according to forward scatter factor (FSC-A) and side scatter factor (SSC-A). Left panel: The

percentage of cells corresponding to the fraction of cells with the highest size and granularity is shown. Right panel: Morphology of NHDFs at the different PDs. Bars represent 100 µm. This experiment is representative of 3 independent ones. (**C**) Diameter of NHDFs in suspension at different population doublings. Cells were counted in 3 independent microscopic fields. The bar chart represents the mean ± SD of each 3 counts. The results are representative of 2 independent experiments. (**D**) Percentage of SA-β-Gal-positive cells in exponentially growing or senescent NHDFs. SA-β-Gal positive cells were counted in 3 independent microscopic fields. The bar chart represents the mean ± SD of each 3 counts. The results are representative of 3 independent experiments. (**E**) Western-blot analysis of 16 and p53 protein levels in total protein extracts from exponentially growing or senescent NHDFs. GAPDH was used as loading control. The bands were quantified using Image J software. The results are given as a ratio to GAPDH.

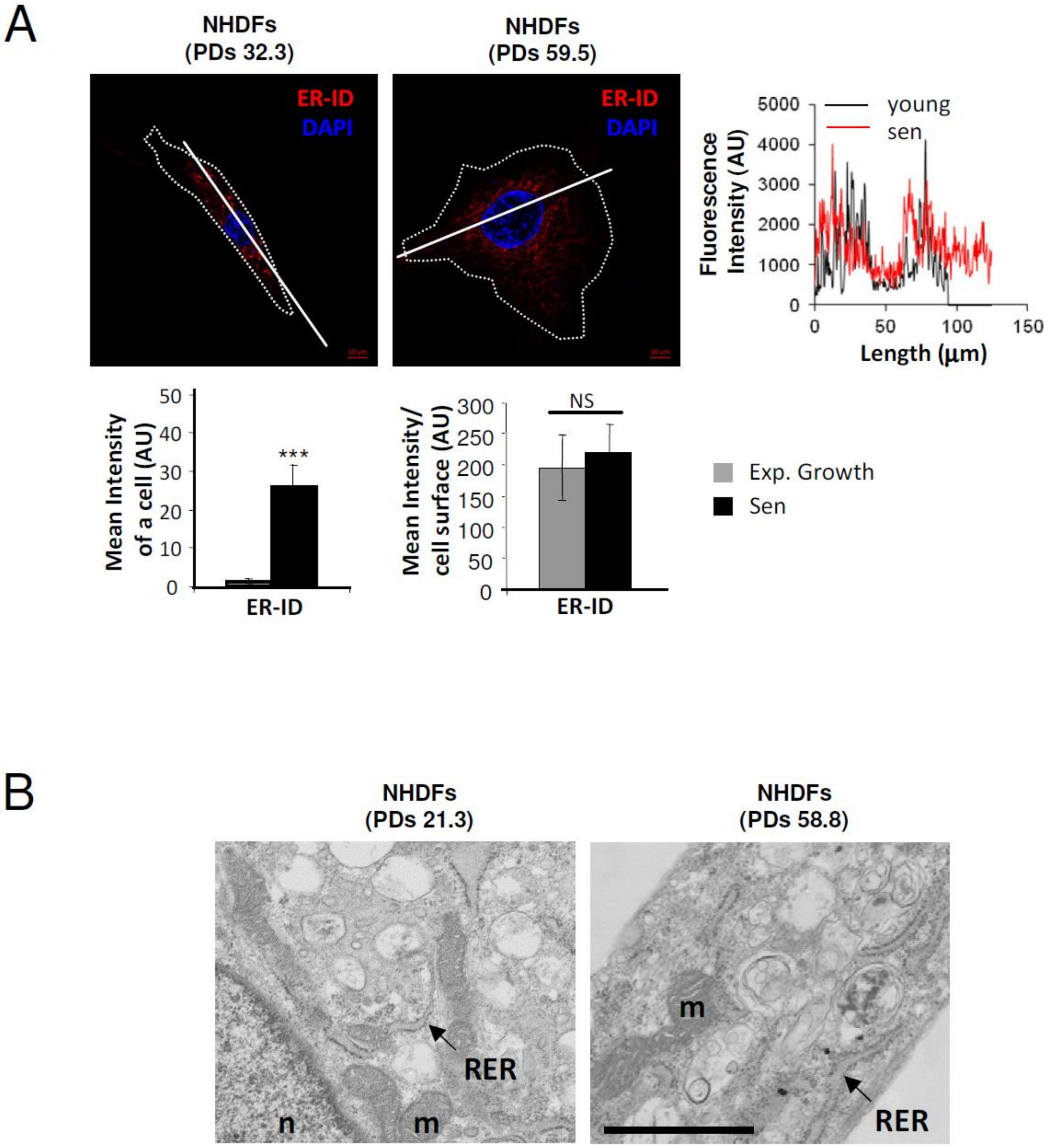

Supplementary Figure S3: ER expands during replicative senescence of NHDFs but is not structurally modified. (**A**) Upper panels: exponentially growing (PDs 32.3) and senescent NHDFs (PDs 59.5) were seeded onto coverslips, fixed, and processed for fluorescence detection of ER using the ER-ID dye (*red*). Cell nuclei were detected by DAPI staining (*blue*). Each condition was tested in triplicate; representative images are shown. Bars represent 10 μm. The graph (right panel) shows the distribution of fluorescence intensity (arbitrary units) of representative growing and senescent NHDFs along the largest cellular axis (white line in left panels). Lower panels: Quantification of the fluorescence signal

coupled to ER-ID, as mean intensity of a cell, and mean intensity/cell surface, using Image J software. Values are means of >40 cells ± SD. This experiment is representative of 3 independent ones. (**B**) Ultrastructure of exponentially growing (PDs 21.3) and senescent NHDFs (PDs 58.8) by TEM analysis. n: nucleus, RER: rough endoplasmic reticulum, m: mitochondria. (Bar =1 μm).

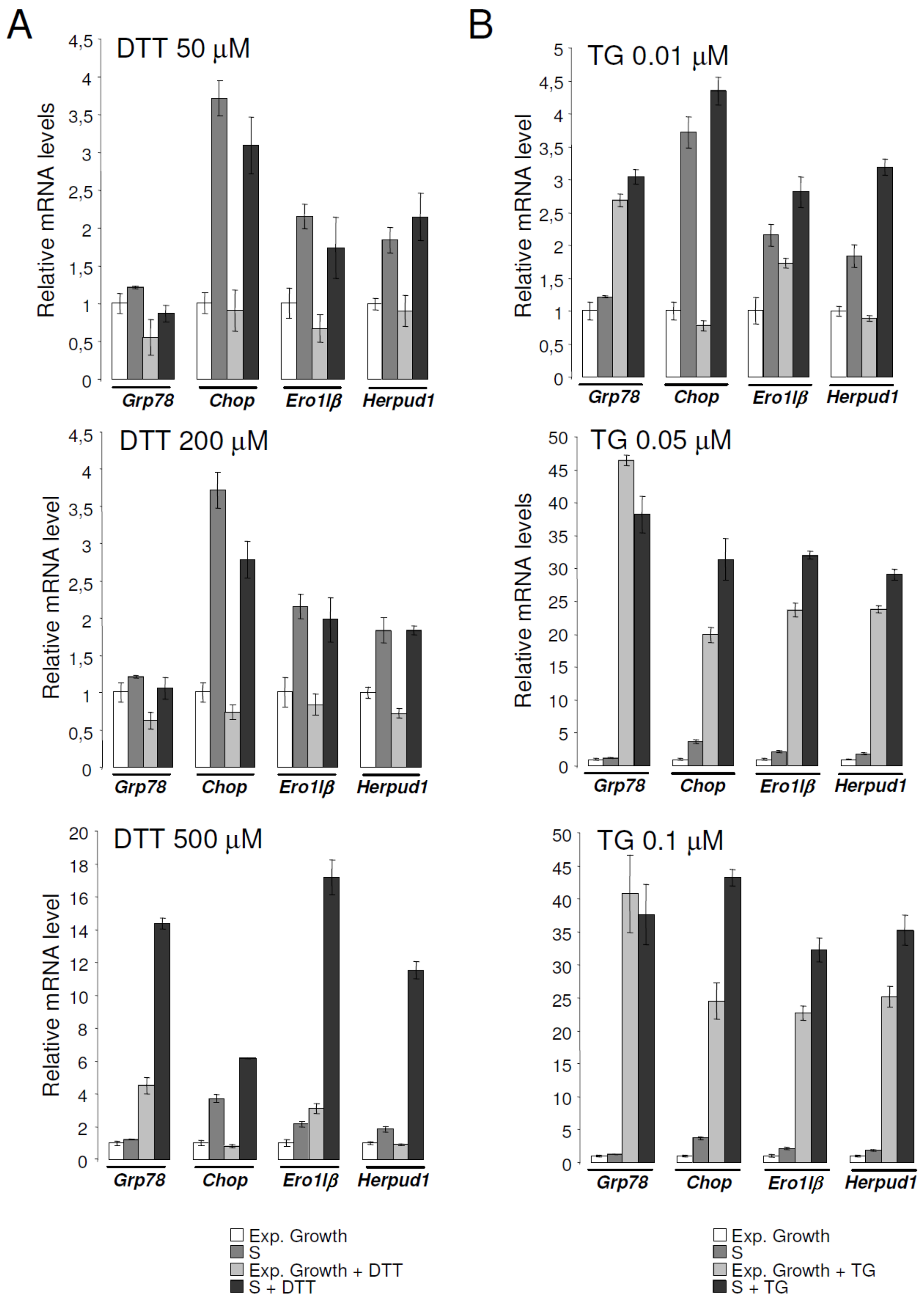

Supplementary Figure S4: Senescent NHDFs retain their ability to respond to acute ER stress. (**A**) Exponentially growing and senescent NHDFs were treated with DTT (50, 200 or 500 μM) for 16 hrs. (**B**) Exponentially growing and senescent NHDFs were treated with TG

(0.01, 0.05 or 0.1 μM) for 16 hrs. In both treatments, UPR target genes mRNA levels were measured by qRT-PCR and were normalized to EAR levels. Measures were performed in triplicate. Results are shown as means ± SD of the 3 measures. .

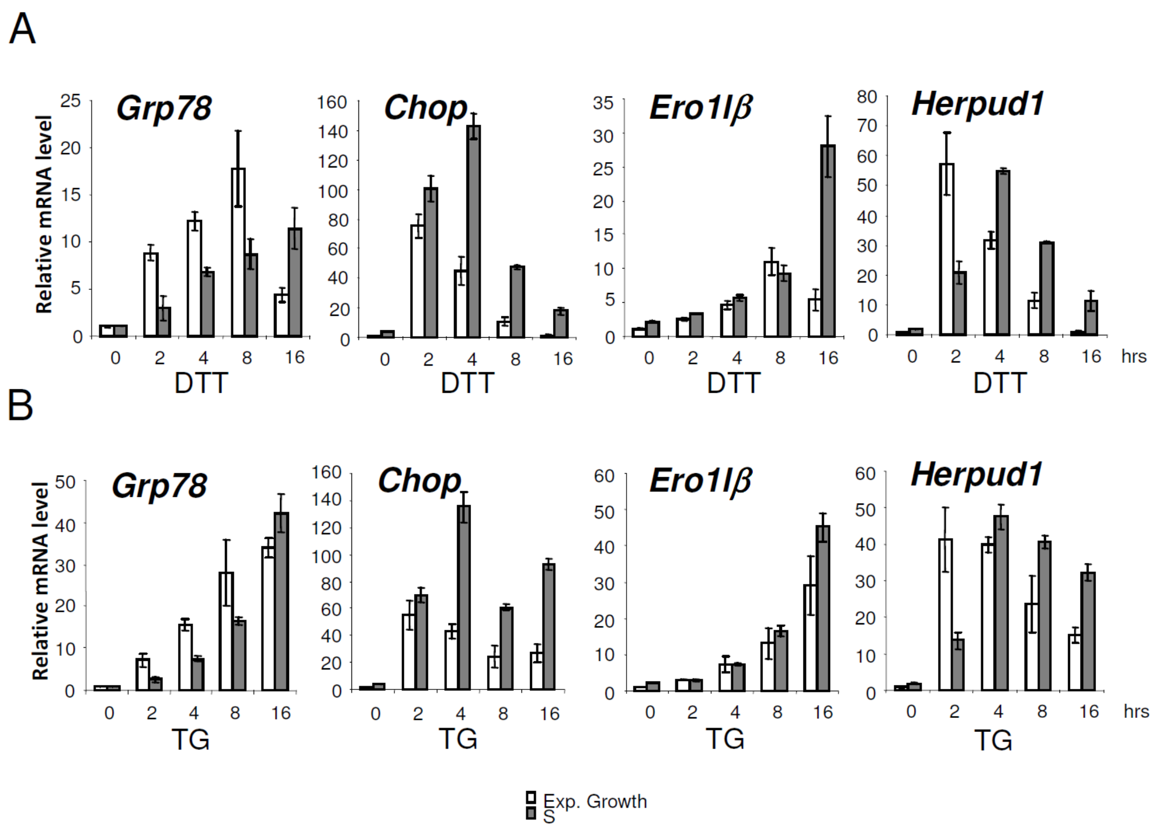

Supplementary Figure S5: The response to acute ER stress is delayed in senescent NHDFs. (**A**) Exponentially growing and senescent NHDFs were treated with DTT 500 µM for the indicated times. (**B**) Exponentially growing and senescent NHDFs were treated with TG 0.1 µM for the indicated times. In both cases, UPR target genes mRNA levels were measured by qRT-PCR and were normalized to EAR levels. Measures were performed in triplicate. Results are shown as mean fold induction ± SD of ER stressors-treated cells vs untreated cells.

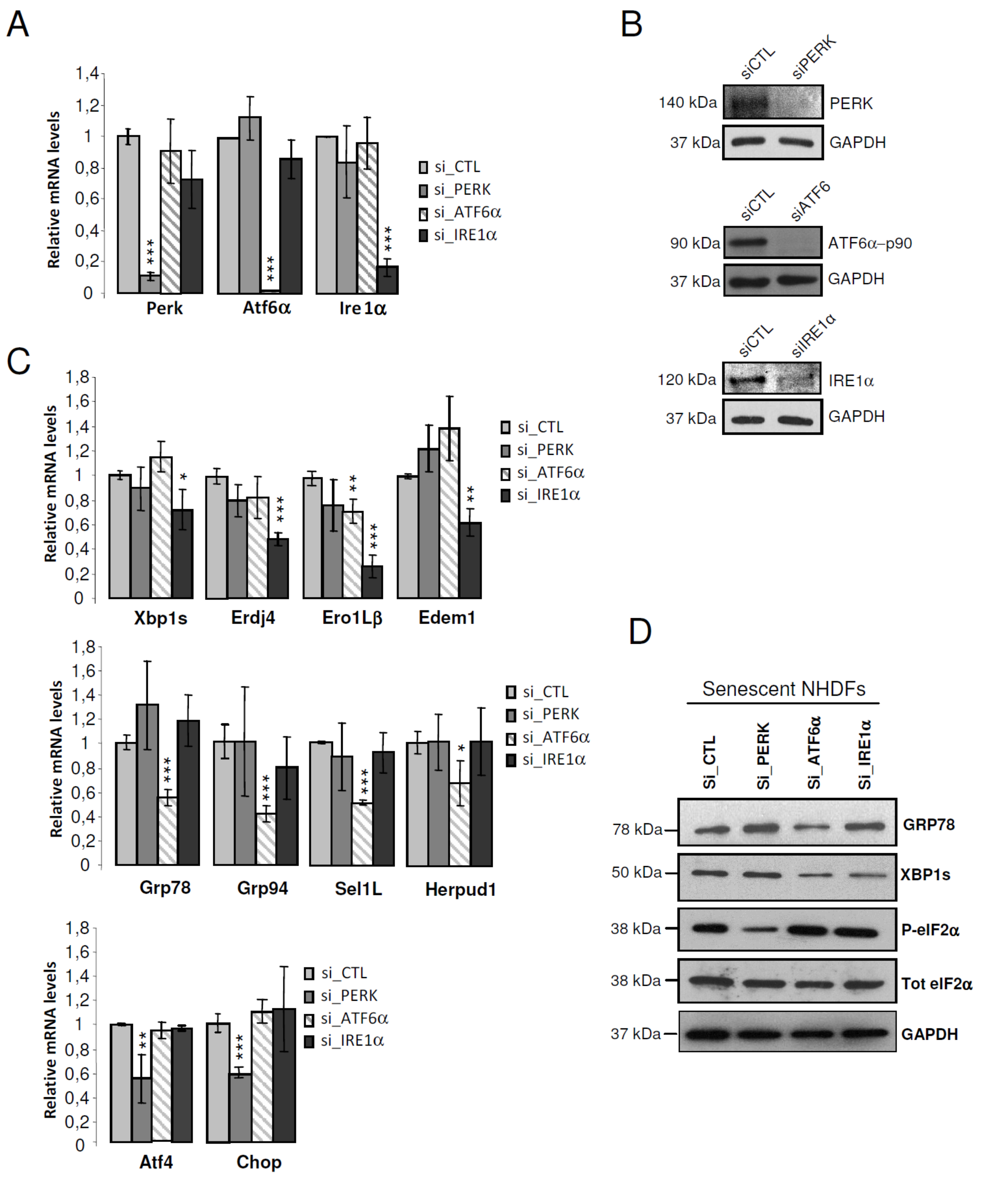

Supplementary Figure S6: Evaluation of PERK, ATF6α, IRE1α silencing efficiency in senescent NHDFs. (**A**) Analysis of PERK, ATF6α and IRE1α mRNA levels by qRT-PCR in senescent NHDFs subjected to individual PERK, ATF6α or IRE1α silencing compared to a non-target siRNA as control 96h after transfection. The results were normalized to EAR and represent mean ± SD of three independent experiments. (**B**) Analysis of PERK, ATF6α, IRE1α protein levels in senescent NHDFs subjected to individual PERK, ATF6α or IRE1α

silencing compared to a non-target siRNA as control 96h after transfection. GAPDH was used as loading control. (**C**) IRE1α-target gene (Xbp1s, Erdj4, Ero1lβ, Edem1), ATF6α-target genes (Grp78, Grp94, Sel1L, Herpud1), and PERK-target genes (Atf4 and Chop), mRNA levels were measured by qRT-PCR and were normalized to EAR levels. Measures were performed in triplicate. The bar charts represent the mean ± SD. (**D**) Senescent NHDFs were treated as in (A), total protein extracts were analyzed by immunoblotting with anti-GRP78, anti-Phospho-eIF2α and anti-XBP1s antibodies. Expression of total eIF2α and GAPDH was used as loading controls.

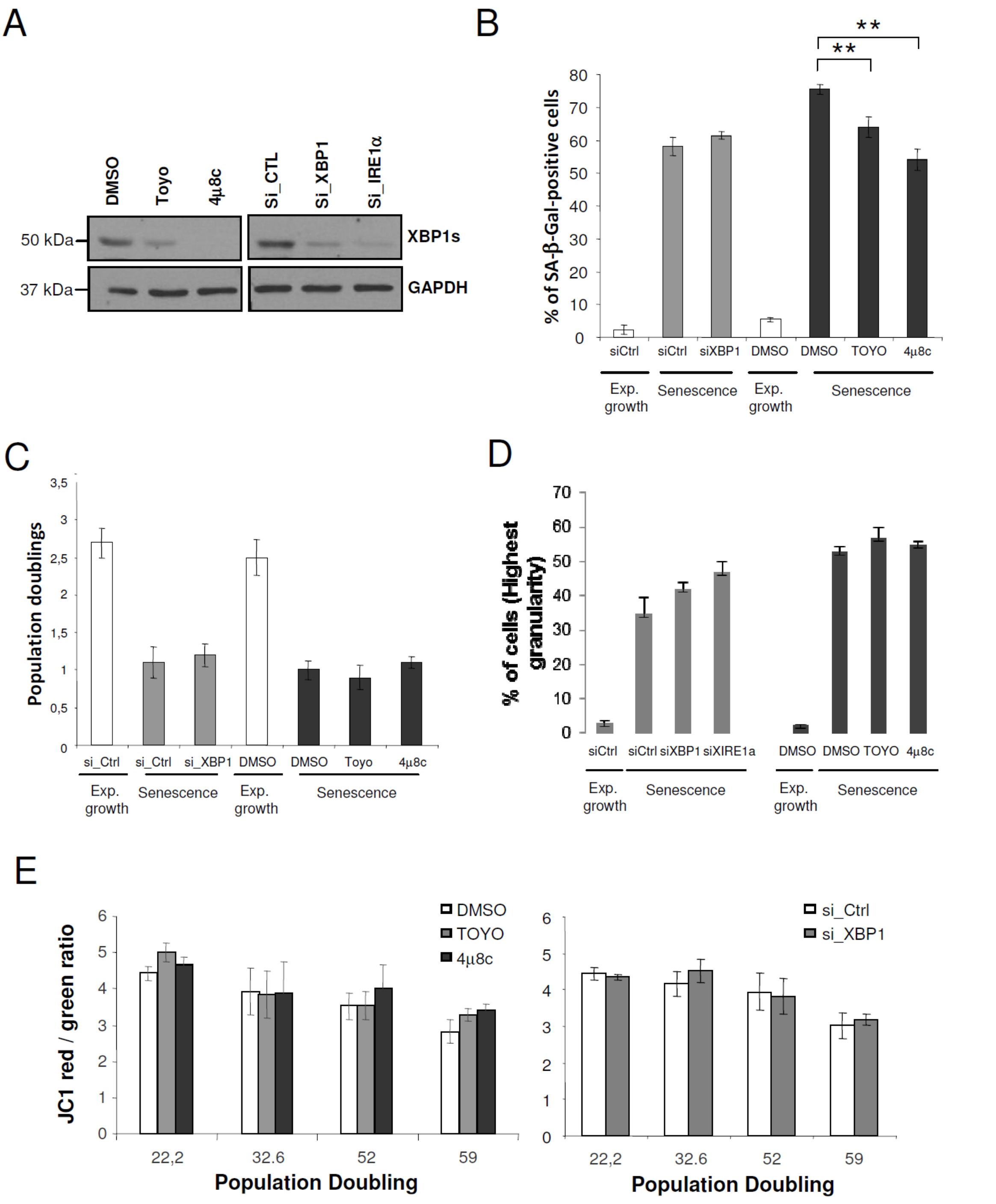

Supplementary Figure S7: Impact of the IRE1/XBP1 axis on the senescence markers in NHDFs. (A) Analysis of XBP1s protein levels in senescent NHDFs subjected to toyocamycin (5 nM) or 4µ8c (10 µM) treatments for 4 days or subjected to individual XBP1 or IRE1α silencing compared to a non-target siRNA as control. GAPDH was used as loading control. **(B)** Exponentially growing or senescent NHDFs were subjected to XBP1 silencing by siRNA as well as a non-target control or treatment with toyocamycin (5 nM) or 4µ8c (10 µM) or

vehicle (DMSO). The percentage of SA-β-Gal-positive cells 4 days after the siRNA transfection or after drug treatments is indicated. SA-β-Gal positive cells were counted in 3 independent microscopic fields. The bar chart represents the mean ± SD of each 3 counts. This experiment is representative of 2 independent ones. **(C)** Exponentially growing or senescent NHDFs were treated as in B. Cells were counted 4 days after the siRNA transfection or after drug treatments and the population doublings were calculated. The bar chart represents the mean ± SD of the count of three independent culture dishes. **(D)** NHDFs were treated as in (B), counted 4 days after the siRNA transfection or treatments, and subjected to flow cytometry analysis to assess their side scatter factor (SSC-A) value representative of their granularity content. The percentage of cells in the subpopulation with the highest granularity was measured in triplicate. The bar chart represents the mean ± SD of each 3 measures. The results are representative of 3 independent experiments. **(E)** NHDFs at different population doubling (as indicated) were treated as in (B), and stained with JC-1. Bar chart representing the JC-1 red/green ratio is given. This experiment represents the mean ± SD of 3 independent ones.

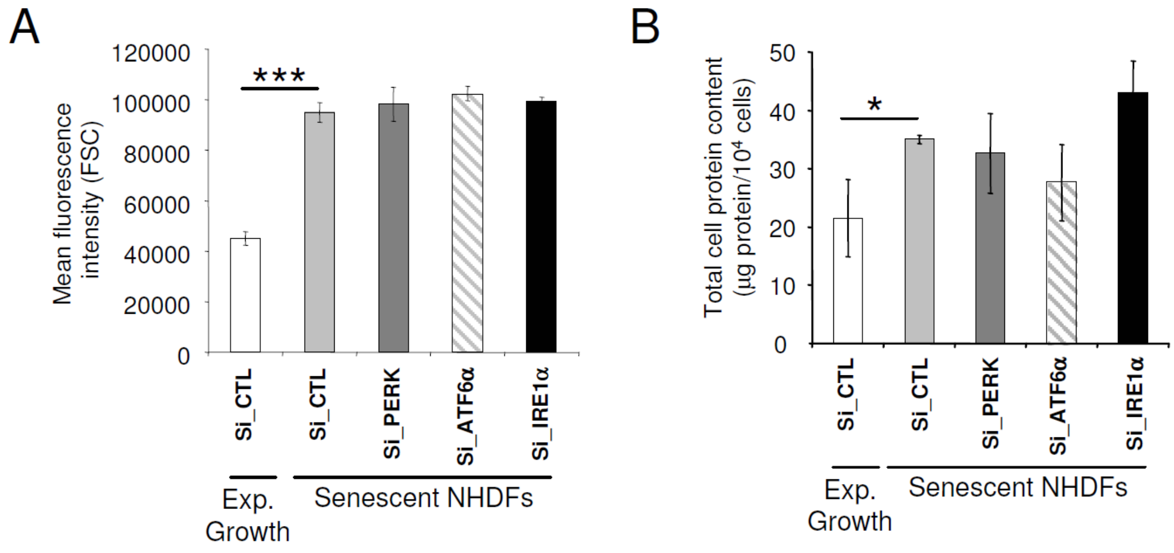

Supplementary Figure S8: The UPR does not control the increased size and protein content at senescence. (A) Exponentially growing and senescent NHDFs were subjected to PERK, ATF6α or IRE1α silencing by siRNA as well as a non-target control. Four days after the siRNA transfection, cells were subjected to flow cytometry analysis to assess their forward scatter factor (FSC-A) value representative of their size. The bar chart represents the mean ± SD of 3 independent measures. **(B)** Exponentially growing and senescent NHDFs were subjected to PERK, ATF6α or IRE1α silencing by siRNA as well as a non-target control. The total protein concentration was determined by the bicinchoninic acid method. Data are presented as mean ± SD of 3 independent experiments.

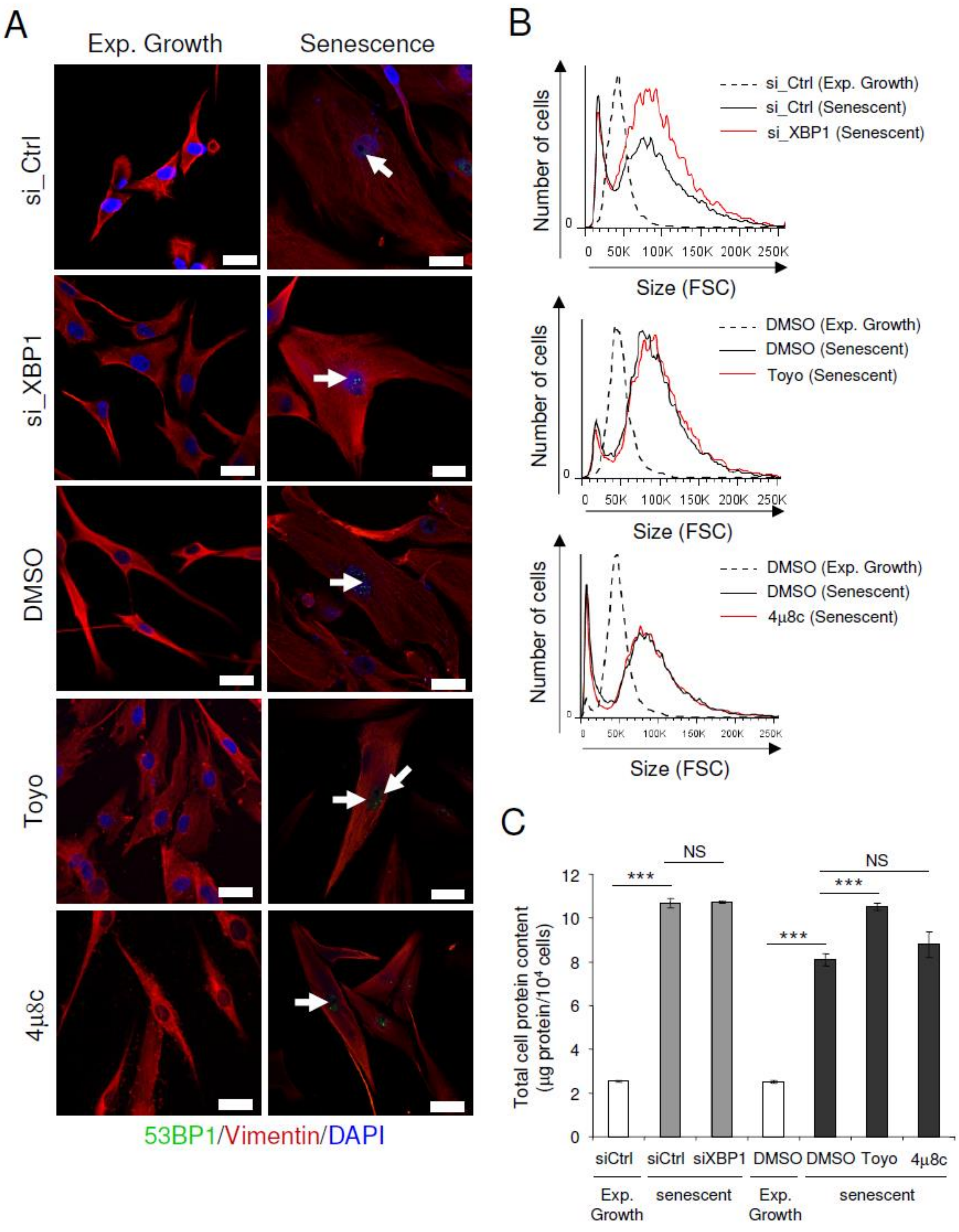

Supplementary Figure S9: Impact of the IRE1/XBP1 axis on the senescence-associated morphological changes. (**A**) Exponentially growing and senescent NHDFs were subjected to XBP1 silencing by siRNA as well as a non-target control or treated with the IRE1α pharmacological inhibitors toyocamycin (5 nM) or 4µ8c (10 µM). Immunofluorescence analysis was performed on endogenous vimentin (*red*), 53BP1 *(green)*, and cell nuclei were

detected by DAPI staining (*blue*). Each condition was tested in triplicate; representative images are shown. Bars represent 100 μm. (**B**) Exponentially growing and senescent NHDFs were treated as in (A) and were subjected to flow cytometry analysis. Cell distribution was plotted against cell size (forward scattering, FSC). (**C**) NHDFs were treated as in (A) and the total protein concentration was determined by the bicinchoninic acid method. Data are presented as mean ± SD of 3 independent experiments.

Supplemental Tables

Upregulated genes

Name	Gene Symbol	Gene name	logFC	adj,P,Val
NM_173512	SLC38A11	solute carrier family 38, member 11	4,72871	8,41E-09
NM_019885	CYP26B1	cytochrome P450, family 26, subfamily B, polypeptide 1	4,16449	8,41E-09
NR_038981	LINC01013	long intergenic non-protein coding RNA 1013	4,09087	2,65E-08
NM_003026	SH3GL2	SH3-domain GRB2-like 2	3,88112	2,42E-08
NM_001004439	ITGA11	integrin, alpha 11	3,70159	1,98E-07
NR_028334	KRT18P55	keratin 18 pseudogene 55	3,58586	8,41E-09
NM_003719	PDE8B	phosphodiesterase 8B	3,57026	7,25E-08
NM_004482	GALNT3	UDP-N-acetyl-alpha-D-galactosamine:polypeptide N-acetylgalactosaminyltransferase 3	3,50051	9,77E-07
NM_000224	KRT18	keratin 18	3,48614	2,42E-08
NM_021219	JAM2	junctional adhesion molecule 2	3,1931	2,26E-07
ENST00000480456	Atp5j	ATP synthase, H+ transporting, mitochondrial F0 complex, subunit F6	3,11799	7,56E-06
NM_001001547	CD36	CD36 molecule (thrombospondin receptor)	3,11153	2,17E-08
NM_001792	CDH2	cadherin 2, type 1, N-cadherin (neuronal)	2,97037	3,99E-08
NM_198404	KCTD4	potassium channel tetramerisation domain containing 4	2,87568	1,85E-06
NM_001122679	Odz2	odz, odd Oz/ten-m homolog 2 (Drosophila)	2,83654	1,83E-07
NM_005639	SYT1	synaptotagmin I	2,80026	1,13E-07
NM_198148	CPXM2	carboxypeptidase X (M14 family), member 2	2,76445	3,28E-08
NM_001432	EREG	epiregulin	2,71608	1,78E-07
NM_172037	RDH10	retinol dehydrogenase 10 (all-trans)	2,70115	3,92E-08
NM_153350	FBXL16	F-box and leucine-rich repeat protein 16	2,69881	5,96E-07
NM_032578	MYPN	myopalladin	2,69345	1,88E-07
NM_002245	KCNK1	potassium channel, subfamily K, member 1	2,67404	3,99E-08
NM_002240	KCNJ6	potassium inwardly-rectifying channel, subfamily J, member 6	2,67038	3,53E-07
NM_006273	CCL7	chemokine (C-C motif) ligand 7	2,6073	1,91E-06
NM_004319	ASTN1	astrotactin 1	2,59525	4,88E-08
NM_139248	LIPH	lipase, member H	2,53829	2,30E-06
NM_001146037	Slc14a1	solute carrier family 14 (urea transporter), member 1 (Kidd blood group)	2,50135	2,75E-07
NM_153225	RPESP	RPE-spondin	2,49402	3,28E-08
NM_001337	CX3CR1	chemokine (C-X3-C motif) receptor 1	2,4757	3,77E-05
NM_002279	KRT33B	keratin 33B	2,46209	8,75E-08
NM_018334	LRRN3	leucine rich repeat neuronal 3	2,43948	1,78E-07
NM_014505	KCNMB4	potassium large conductance calcium-activated channel, subfamily M, beta member 4	2,41629	3,28E-08
NM_007034	DNAJB4	DnaJ (Hsp40) homolog, subfamily B, member 4	2,38914	5,70E-08
NM_172069	PLEKHH2	pleckstrin homology domain containing, family H (with MyTH4 domain) member 2	2,37933	4,22E-06
NM_175887	PRR15	proline rich 15	2,37666	1,43E-07
NM_001001557	GDF6	growth differentiation factor 6	2,37343	4,57E-07
NM_182904	P4HA3	procollagen-proline, 2-oxoglutarate 4-dioxygenase, alpha polypeptide III	2,33289	3,89E-07
NM_000576	IL1B	interleukin 1, beta	2,32038	1,12E-07
NM_001025252	TPD52	tumor protein D52	2,31536	1,88E-07
NM_000014	A2M	alpha-2-macroglobulin	2,31318	3,89E-08
NM_000507	FBP1	fructose-1,6-bisphosphatase 1	2,31292	2,48E-06
NM_031958	KRTAP3-1	keratin associated protein 3-1	2,28931	4,95E-07
NM_014905	GLS	glutaminase	2,27353	1,27E-07
NM_001135599	TGFB2	transforming growth factor, beta 2	2,21832	3,68E-06
NM_030967	KRTAP1-1	keratin associated protein 1-1	2,21762	2,30E-06
NM_033400	ZFHX2	zinc finger homeobox 2	2,19111	1,81E-06
NM_003014	SFRP4	secreted frizzled-related protein 4	2,15732	2,04E-07
NM_080874	ASB5	ankyrin repeat and SOCS box-containing 5	2,15027	1,27E-07
NM_207334	FAM43B	family with sequence similarity 43, member B	2,14936	1,93E-07
NM_033211	C5orf30	chromosome 5 open reading frame 30	2,13658	1,38E-07
NM_001080471	Pear1	platelet endothelial aggregation receptor 1	2,13063	9,23E-07
NM_014399	TSPAN13	tetraspanin 13	2,10136	3,74E-07
NM_144646	IGJ	immunoglobulin J polypeptide, linker protein for immunoglobulin alpha and mu polypeptides	2,09694	6,05E-06
NM_152721	DOK6	docking protein 6	2,09647	3,74E-07
NM_003947	KALRN	kalirin, RhoGEF kinase	2,09452	7,35E-06
NM_018650	MARK1	MAP/microtubule affinity-regulating kinase 1	2,08761	1,85E-07
NM_002982	CCL2	chemokine (C-C motif) ligand 2	2,04929	8,53E-07
NM_001080416	mybl1	v-myb myeloblastosis viral oncogene homolog (avian)-like 1	2,0462	1,65E-07
NM_001993	F3	coagulation factor III (thromboplastin, tissue factor)	2,00708	1,13E-07
NM_000147	FUCA1	fucosidase, alpha-L- 1	2,00614	1,36E-07
NM_021572	ENPP5	ectonucleotide pyrophosphatase/phosphodiesterase 5 (putative function)	1,98566	4,84E-06
NM_203447	DOCK8	dedicator of cytokinesis 8	1,96278	2,01E-06
NM_001409	MEGF6	multiple EGF-like-domains 6	1,95392	9,16E-08
NM_014936	ENPP4	ectonucleotide pyrophosphatase/phosphodiesterase 4 (putative function)	1,94757	5,53E-06
NM_004975	KCNB1	potassium voltage-gated channel, Shab-related subfamily, member 1	1,93799	5,56E-07
NM_002589	PCDH7	BH-protocadherin (brain-heart)	1,9097	1,57E-05
NM_053056	CCND1	cyclin D1	1,90328	1,07E-06
NM_003526	HIST1H2BC	histone cluster 1, H2bc	1,88983	7,83E-07
NM_001172173	CSRNP3	cysteine-serine-rich nuclear protein 3	1,88475	4,07E-06
NR_038387	PA2G4	roliferation-associated 2G4, 38kDa	1,87933	5,04E-07
NM_001040874	HIST2H2AA4	histone cluster 2, H2aa4	1,87227	5,36E-07
NM_007029	STMN2	stathmin-like 2	1,8677	3,43E-07
THC2550342	CTSB	Cathepsin B	1,85867	2,08E-07
ENST00000314088	Hist1h2ac	histone cluster 1, H2ac	1,85621	1,88E-07
NM_032211	LOXL4	lysyl oxidase-like 4	1,85161	4,77E-07
NM_002426	MMP12	matrix metallopeptidase 12 (macrophage elastase)	1,83803	2,54E-07
NM_001901	CTGF	connective tissue growth factor	1,83156	8,85E-07
NM_001850	COL8A1	collagen, type VIII, alpha 1	1,81319	1,03E-07

NM_000963	PTGS2	prostaglandin-endoperoxide synthase 2	1,79039	7,20E-07
NM_000930	PLAT	plasminogen activator, tissue	1,78747	1,19E-07
NM_016341	PLCE1	phospholipase C, epsilon 1	1,78226	3,89E-07
NM_025049	PIF1	PIF1 5'-to-3' DNA helicase homolog (S. cerevisiae)	1,77956	5,99E-06
NM_016352	CPA4	carboxypeptidase A4	1,77382	3,39E-05
AA837799	TIMP3	TIMP metallopeptidase inhibitor 3	1,76856	2,52E-06
NM_004796	NRXN3	neurexin 3	1,76078	0,0004598
NM_019029	CPVL	carboxypeptidase, vitellogenic-like	1,75711	2,21E-05
NM_181361	KCNMB2	potassium large conductance calcium-activated channel, subfamily M, beta member 2	1,75376	6,72E-06
NM_032623	OSAP	ovary-specific acidic protein	1,74752	1,27E-07
NM_207481	NCKAP5	NCK-associated protein 5	1,74148	2,36E-06
NM_000210	ITGA6	integrin, alpha 6	1,73559	1,78E-07
NM_021065	HIST1H2AD	histone cluster 1, H2ad	1,73492	1,09E-07
NM_173508	SLC35F3	solute carrier family 35, member F3	1,72772	7,16E-06
NM_001080826	SGK223	homolog of rat pragma of Rnd2	1,71671	5,91E-07
NM_000602	SERPINE1	serpin peptidase inhibitor, clade E, member 1	1,71448	2,04E-06
NM_002561	P2RX5	purinergic receptor P2X, ligand-gated ion channel, 5	1,71075	1,11E-06
NM_198449	EMB	embigin	1,70278	1,97E-06
NM_198461	LONRF2	LON peptidase N-terminal domain and ring finger 2	1,68436	4,14E-05
NM_020130	C8orf4	chromosome 8 open reading frame 4	1,68165	2,35E-06
NM_018076	ARMC4	armadillo repeat containing 4	1,68071	7,71E-07
NM_003530	HIST1H3D	histone cluster 1, H3d	1,67132	0,0001189
NM_003629	PIK3R3	phosphoinositide-3-kinase, regulatory subunit 3 (p55, gamma)	1,67002	0,0001851
NM_003633	ENC1	ectodermal-neural cortex (with BTB-like domain)	1,65917	2,01E-06
NM_003480	MFAP5	microfibrillar associated protein 5	1,64434	3,82E-07
NM_052907	TMEM132B	transmembrane protein 132B	1,63991	4,16E-05
NM_001257	CDH13	cadherin 13, H-cadherin (heart)	1,63862	8,43E-07
NM_020445	ACTR3B	ARP3 actin-related protein 3 homolog B (yeast)	1,62964	1,77E-06
NM_024893	SYNDIG1	synapse differentiation inducing 1	1,62915	1,82E-05
NM_021102	SPINT2	serine peptidase inhibitor, Kunitz type, 2	1,62613	3,94E-07
NM_000820	GAS6	growth arrest-specific 6	1,62448	4,07E-06
NM_005408	CCL13	chemokine (C-C motif) ligand 13	1,61991	0,0001791
NM_025244	TSGA10	testis specific, 10	1,61715	1,67E-05
NM_001144995	CCDC85C	coiled-coil domain containing 85C	1,61539	3,19E-07
NM_080593	HIST1H2BK	histone cluster 1, H2bk	1,61521	5,90E-07
NM_001759	CCND2	cyclin D2	1,6128	9,23E-07
NR_038366	HOTAIRM1	HOXA transcript antisense RNA, myeloid-specific 1	1,6072	1,29E-06
NM_001007189	IGIP	IgA-inducing protein	1,60113	1,09E-05
NM_003918	GYG2	glycogenin 2	1,59564	1,50E-06
NM_006379	SEMA3C	sema domain, immunoglobulin domain (Ig), short basic domain, secreted, (semaphorin) 3C	1,5914	3,35E-07
NM_000425	L1CAM	L1 cell adhesion molecule	1,59122	5,50E-06
NM_020116	FSTL5	follistatin-like 5	1,58378	8,86E-06
NM_152913	TMEM130	transmembrane protein 130	1,57991	1,03E-07
	krt8	keratin 8 pseudogene 9; similar to keratin 8; keratin 8	1,57927	9,44E-06
NM_015973	GAL	galanin	1,57089	1,43E-07
NM_002517	NPAS1	neuronal PAS domain protein 1	1,56104	8,22E-06
AK130878	C8Orf84	Chromosome 8 open reading frame 84	1,56052	0,0001192
NM_003486	SLC7A5	solute carrier family 7 (cationic amino acid transporter, y+ system), member 5	1,55672	1,48E-06
NM_133494	NEK7	NIMA (never in mitosis gene a)-related kinase 7	1,55	0,0003315
NM_031938	BCDO2	beta-carotene dioxygenase 2	1,54988	1,44E-05
NM_052958	C8orf34	chromosome 8 open reading frame 34	1,52841	6,98E-07
NM_007193	ANXA10	annexin A10	1,51634	9,64E-06
NM_003522	HIST1H2BF	histone cluster 1, H2bf	1,51412	1,44E-07
NM_021058	HIST1H2BJ	histone cluster 1, H2bj	1,51355	1,04E-06
NM_001165252	KRTAP2-3	keratin associated protein 2-1	1,50815	6,05E-06
NM_017551	GRID1	glutamate receptor, ionotropic, delta 1	1,50514	3,13E-05
NM_022135	POPDC2	popeye domain containing 2	1,50481	4,72E-07

Downregulated genes

Name	Gene Symbol	Gene Name	logFC	adj,P,Val
NM_001615	ACTG2	Actin, gamma 2, smooth muscle, enteric	-4,04114	8,41E-09
NM_207328	GPAT2	glycerol-3-phosphate acyltransferase 2, mitochondrial	-3,67824	3,99E-08
NM_199168	CXCL12	Chemokine (C-X-C motif) ligand 12 (stromal cell-derived factor 1)	-3,53595	8,67E-09
NM_000349	STAR	Steroidogenic acute regulatory protein	-3,38093	2,56E-07
NM_153000	APCDD1	Adenomatosis polyposis coli down-regulated 1	-3,04316	5,36E-08
NM_000475	NR0B1	Nuclear receptor subfamily 0, group B, member 1	-3,00187	2,21E-07
NM_014799	HEPH	Hephaestin	-2,98099	1,51E-06
NM_003613	CILP	Cartilage intermediate layer protein, nucleotide pyrophosphohydrolase	-2,8796	3,12E-07
NM_033260	FOXQ1	Forkhead box Q1	-2,76262	1,44E-07
NM_003391	WNT2	Wingless-type MMTV integration site family member 2	-2,65996	8,75E-08
NM_006308	HSPB3	Heat shock 27kDa protein 3	-2,64266	2,17E-08
NM_014033	METTL7A	Methyltransferase like 7A	-2,60934	9,72E-08
NM_004484	GPC3	Glypican 3	-2,53508	7,47E-07
NM_004102	FABP3	fatty acid binding protein 3, muscle and heart (mammary-derived growth inhibitor)	-2,48196	5,80E-08
NM_000667	ADH1A	alcohol dehydrogenase 1A (class I), alpha polypeptide	-2,47433	3,09E-07
NM_015507	EGFL6	EGF-like-domain, multiple 6	-2,46102	0,0001942
NM_030915	LBH	Limb bud and heart development homolog (mouse)	-2,433	2,21E-07
NM_005940	MMP11	Matrix metallopeptidase 11 (stromelysin 3)	-2,43192	1,03E-07
NM_178565	RSPO2	R-spondin 2 homolog (Xenopus laevis)	-2,36018	2,59E-06
NM_005063	SCD	Stearoyl-CoA desaturase (delta-9-desaturase)	-2,30634	1,44E-07

NM_024574	C4orf31	Chromosome 4 open reading frame 31	-2,30296	1,20E-05
NM_032333	C10orf58	Chromosome 10 open reading frame 58	-2,27727	1,86E-07
NM_033342	TRIM7	Tripartite motif-containing 7	-2,24619	1,88E-07
NM_181078	IL21R	Interleukin 21 receptor	-2,22257	2,42E-05
NM_006475	POSTN	Periostin, osteoblast specific factor	-2,17098	1,88E-07
NM_024812	BAALC	Brain and acute leukemia, cytoplasmic	-2,16754	2,15E-06
NM_078626	CDKN2C	Cyclin-dependent kinase inhibitor 2C (p18, inhibits CDK4)	-2,12094	2,22E-07
NM_016563	RASL12	RAS-like, family 12	-2,07482	1,50E-05
NM_000612	IGF2	Insulin-like growth factor 2 (somatomedin A)	-2,04372	4,54E-07
NM_004794	RAB33A	RAB33A, member RAS oncogene family	-2,00836	1,43E-07
NM_001511	CXCL1	Chemokine (C-X-C motif) ligand 1 (melanoma growth stimulating activity, alpha)	-2,00486	3,64E-07
NM_003739	AKR1C3	Aldo-keto reductase family 1, member C3	-2,00424	1,16E-07
NM_152304	RAB42	RAB42, member RAS oncogene family	-1,99928	6,95E-07
NM_000955	PTGER1	Prostaglandin E receptor 1 (subtype EP1), 42kDa	-1,96053	4,83E-06
NM_004098	EMX2	Empty spiracles homeobox 2	-1,93948	1,15E-07
NM_004626	WNT11	Wingless-type MMTV integration site family, member 11	-1,93355	8,45E-05
NM_006546	IGF2BP1	Insulin-like growth factor 2 mRNA binding protein 1	-1,92512	7,28E-08
NM_000669	ADH1C	alcohol dehydrogenase 1C (class I), gamma polypeptide	-1,92385	7,97E-06
NM_001710	CFB	Complement factor B	-1,91909	1,62E-05
NM_015714	G0S2	G0/G1switch 2	-1,8895	4,07E-06
NM_001645	APOC1	Apolipoprotein C-I	-1,88845	9,78E-07
NM_001099	ACPP	Acid phosphatase, prostate	-1,88577	1,32E-05
NM_001185095	AIF1L	Allograft inflammatory factor 1-like	-1,88414	1,83E-06
NM_020675	SPC25	SPC25, NDC80 kinetochore complex component, homolog (S. cerevisiae)	-1,88339	1,91E-06
NM_032849	C13orf33	chromosome 13 open reading frame 33	-1,87848	2,40E-07
NM_030820	COL21A1	Collagen, type XXI, alpha 1	-1,8739	6,91E-05
NM_033292	CASP1	Caspase 1, apoptosis-related cysteine peptidase (interleukin 1, beta, convertase)	-1,85466	8,19E-08
NM_004934	CDH18	Cadherin 18, type 2	-1,8532	5,42E-07
NM_005357	LIPE	Lipase, hormone-sensitive	-1,85261	2,04E-07
NM_001025100	MBP	Myelin basic protein	-1,84638	6,18E-07
NM_175569	XG	Xg blood group	-1,82898	1,96E-06
NM_018492	PBK	PDZ binding kinase	-1,82075	1,97E-06
NM_004120	GBP2	Guanylate binding protein 2, interferon-inducible	-1,81984	4,07E-06
NM_001205272	KRBOX1	KRAB box domain containing 1	-1,81951	0,0001055
NM_001353	AKR1C1	Aldo-keto reductase family 1, member C1	-1,80829	7,25E-08
NM_019609	CPXM1	carboxypeptidase X (M14 family), member 1	-1,80421	4,25E-06
NM_002133	HMOX1	Heme oxygenase (decycling) 1	-1,79321	6,40E-08
NM_181789	GLDN	Gliomedin	-1,78317	4,53E-05
NM_006547	IGF2BP3	Insulin-like growth factor 2 mRNA binding protein 3	-1,77719	1,17E-06
NM_016343	CENPF	Centromere protein F, 350/400ka (mitosin)	-1,76411	3,12E-07
NM_001040710	C2orf84	Chromosome 2 open reading frame 84	-1,76404	3,96E-05
NM_000867	HTR2B	5-hydroxytryptamine (serotonin) receptor 2B	-1,76123	8,19E-05
NM_032784	RSPO3	R-spondin 3 homolog (Xenopus laevis)	-1,75546	2,62E-05
NM_001928	CFD	Complement factor D (adipsin)	-1,75473	2,99E-07
NM_022346	NCAPG	non-SMC condensin I complex, subunit G	-1,74101	3,05E-07
NM_001311	CRIP1	Cysteine-rich protein 1 (intestinal)	-1,74037	2,27E-06
NM_001080824	TRABD2A	TraB domain containing 2A	-1,72054	4,84E-06
NM_001406	EFNB3	Ephrin-B3	-1,71014	1,04E-06
NM_005733	KIF20A	kinesin family member 20A	-1,70869	2,04E-07
NM_002487	NDN	Necdin homolog (mouse)	-1,70697	3,31E-07
NM_001289	CLIC2	chloride intracellular channel 2	-1,69278	3,23E-06
NM_005192	CDKN3	Cyclin-dependent kinase inhibitor 3	-1,68934	1,62E-07
NM_021076	NEFH	Neurofilament, heavy polypeptide	-1,68522	2,90E-06
NM_021965	PGM5	Phosphoglucomutase 5	-1,68163	3,23E-06
NM_024693	ECHDC3	Enoyl Coenzyme A hydratase domain containing 3	-1,68053	3,96E-07
NM_005244	EYA2	Eyes absent homolog 2 (Drosophila)	-1,67891	2,50E-05
NM_080284	ABCA6	ATP-binding cassette, sub-family A (ABC1), member 6	-1,67197	1,33E-05
NM_032048	EMILIN2	Elastin microfibril interfacer 2	-1,65376	1,52E-07
NM_001143981	CHRDL1	Chordin-like 1	-1,65269	8,25E-06
NM_000877	IL1R1	Interleukin 1 receptor, type I	-1,65132	1,63E-07
NM_001452	FOXF2	Forkhead box F2	-1,65085	1,18E-06
NM_014750	DLGAP5	Discs, large (Drosophila) homolog-associated protein 5	-1,64633	1,03E-05
NM_014178	STXBP6	Syntaxin binding protein 6 (amisyn)	-1,63553	2,27E-07
NM_013402	FADS1	Fatty acid desaturase 1	-1,62737	1,86E-06
NM_202002	FOXM1	Forkhead box M1	-1,62541	4,62E-06
NM_007115	TNFAIP6	Tumor necrosis factor, alpha-induced protein 6	-1,6178	3,35E-05
NM_015071	ARHGAP26	Rho GTPase activating protein 26	-1,61668	1,06E-06
NM_001012271	BIRC5	baculoviral IAP repeat-containing 5 (survivin)	-1,60751	1,16E-07
NM_021785	RAI2	Retinoic acid induced 2	-1,59941	3,18E-05
NM_001005463	EBF3	Early B-cell factor 3	-1,59629	4,07E-06
NM_018131	CEP55	Centrosomal protein 55kDa	-1,58585	2,92E-06
NM_000041	APOE	apolipoprotein E	-1,58461	6,29E-07
NM_018431	DOK5	docking protein 5	-1,57609	1,67E-06
NM_001113207	F11R	F11 receptor	-1,57041	8,35E-07
NM_013363	PCOLCE2	Procollagen C-endopeptidase enhancer 2	-1,57005	1,92E-07
NM_001034	RRM2	Ribonucleotide reductase M2 polypeptide	-1,56994	9,60E-07
NM_006607	PTTG2	pituitary tumor-transforming 2	-1,56766	4,33E-07
NM_001126132	TNNT1	Troponin T type 1 (skeletal, slow)	-1,56697	1,45E-06
NM_000076	CDKN1C	Cyclin-dependent kinase inhibitor 1C (p57, Kip2)	-1,55737	8,45E-07
NM_019013	FAM64A	family with sequence similarity 64, member A	-1,55732	2,39E-07

NR_003674	KGFLP1	Keratinocyte growth factor-like protein 1	-1,55121	2,94E-07
NM_001012507	C6orf173	Chromosome 6 open reading frame 173	-1,55038	1,61E-05
NM_015187	SEL1L3	sel-1 suppressor of lin-12-like 3 (C. elegans)	-1,54702	1,42E-05
NM_004560	ROR2	Receptor tyrosine kinase-like orphan receptor 2	-1,54634	2,76E-06
NM_014729	TOX	Thymocyte selection-associated high mobility group box	-1,54337	2,49E-06
NM_004900	APOBEC3B	apolipoprotein B mRNA editing enzyme, catalytic polypeptide-like 3B	-1,53234	4,07E-06
NM_001040078	LGALS9C	lectin, galactoside-binding, soluble, 9C	-1,52928	1,91E-07
NM_178448	C9orf140	Chromosome 9 open reading frame 140	-1,52865	3,31E-07
NM_198336	INSIG1	Insulin induced gene 1	-1,5277	3,97E-07
NM_003517	HIST2H2AC	Histone cluster 2, H2ac	-1,52734	1,51E-05
NM_080629	COL11A1	Collagen, type XI, alpha 1	-1,52714	9,20E-07
NM_005573	LMNB1	Lamin B1	-1,52532	9,93E-07
NM_002729	HHEX	Hematopoietically expressed homeobox	-1,52232	5,92E-06
NM_003258	TK1	Thymidine kinase 1, soluble	-1,519	1,76E-07
NM_001822	CHN1	chimerin (chimaerin) 1	-1,51578	7,79E-07
NM_001017534	CARD16	Caspase recruitment domain family, member 16	-1,50629	4,08E-06
NM_013227	ACAN	Aggrecan	-1,50254	9,18E-05
NM_148957	TNFRSF19	Tumor necrosis factor receptor superfamily, member 19	-1,50189	2,85E-05

Supplementary Table 1: The senescence signature in NHDFs. List of the 253 genes of the senescence signature.

Supplementary Table 2: Over-represented Gene Ontology terms and functional assignments of the senescence signature in NHDFs. Excel file.

Name	FWD	REV
Perk	5’-ATGCTTTCACGGTCTTGGTC-3’	5’-TCATCCAGCCTTAGCAAACC-3’
Atf6	5'-AGACTGAAGAGCAGGTGAGCAAA-3'	5'-GATGATGAAAAATGGAGCAGCTT-3'
Ire1	5'-CGAAACTTCCTTTTACCATCCC-3'	5'-CGATGACAAAGTCTGCTGCTT-3'
IL-6	5'-AGGAGACTTGCCTGGTGAAA-3’	5'-CAGGGGTGGTTATTGCATCT-3’
Dcr2	5’-CACTGTCTGCTGGGGAACTT-3’	5’-GTTCGTCGTCTTCATCGTCG-3’
Mcp1	5’-CTCGCGAGCTATAGAAGAATCAC-3’	5’-TCAAAACATCCCAGGGGTAGAACT-3’
Chop	5’– CGACTCGCCGAGCTCTGA-3’	5’– GGGACTGATGCTCCCAATTGT-3’
Grp78	5'-CTACTCCTGCGTCGGCGTGT-3'	5'-CGATGAGCCGCTTGGCGT-3'
Atf4	5’-GTTCTCCAGCGACAAGGCTA-3’	5’-ATCCTGCTTGCTGTTGTTGG-3’
Erdj4	5’-CCGATTTTGGCACACCTAAG-3’	5’-GAGGAGCAGCAGTAGTCGGA-3’
Grp94	5’-CTATTCCGCCTTCCTTGTAGC-3’	5’-CCTCTTGGGTCAGCAATTACA-3’
Orp150	5’-GAAGATGCAGAGCCCATTTC-3’	5’-TCTGCTCCAGGACCTCCTAA-3’
Herpud1	5'-TCCTCCTCCTGACGTTGTAAA-3'	5'-TGCTCGCCATCTAGTACATCC-3'
Ero1Lβ	5'-GGTTTAGGAACTGCCCTGAAG-3'	5'-CAACTATTTCCTGTCGGGTGA-3'
Xbp1s	5’-GCAACAGCAAGTGGTAGA-3’	5’-CTGGAGGGGTGACAAC-3’
EAR	5’-GAGGCTGAGGCAGGAGAATCG-3’	5’-GTCGCCCAGGCTGGAGTG-3’

Supplementary Table 3: List of primers for qRT-PCR
